# Buried object characterization by data-driven surrogates and regression-enabled hyperbolic signature extraction

**DOI:** 10.1038/s41598-023-32925-6

**Published:** 2023-04-07

**Authors:** Reyhan Yurt, Hamid Torpi, Ahmet Kizilay, Slawomir Koziel, Anna Pietrenko-Dabrowska, Peyman Mahouti

**Affiliations:** 1 Department of Electrical-Electronic Engineering, Yalova University, Yalova, 77200 Turkey; 2 Department of Electronics and Communication Engineering, Yıldız Technical University, Istanbul, 34220 Turkey; 3 Engineering Optimization & Modeling Center, Department of Technology, Reykjavik University, Menntavegur 1, 102 Reykjavík, Iceland; 4 Faculty of Electronics, Telecommunications and Informatics, Gdansk University of Technology, Narutowicza 11/12, 80-233 Gdańsk, Poland; 5 Department of Avionics, Yıldız Technical University, Istanbul, 34220 Turkey

**Keywords:** Electrical and electronic engineering, Computational science

## Abstract

This work addresses artificial-intelligence-based buried object characterization using FDTD-based electromagnetic simulation toolbox of a Ground Penetrating Radar (GPR) to generate B-scan data. In data collection, FDTD-based simulation tool, gprMax is used. The task is to estimate geophysical parameters of a cylindrical shape object of various radii, buried at different positions in the dry soil medium simultaneously and independently of each other. The proposed methodology capitalizes on a fast and accurate data-driven surrogate model developed for object characterization in terms of its vertical and lateral position, and the size. The surrogate is constructed in a computationally efficient manner as compared to methodologies using 2D B-scan image. This is achieved by operating at the level of hyperbolic signatures extracted from the B-scan data through linear regression, which effectively reduces the dimensionality and the size of data. The proposed methodology relies on reducing of 2D B-scan image to 1D data including variation of reflected electric fields’ amplitudes with respect to the scanning aperture. The input of the surrogate model is the extracted hyperbolic signature obtained through linear regression executed on the background subtracted B-scan profiles. The hyperbolic signatures encode information about the geophysical parameters of the buried object, including depth, lateral position, and radius, all of which can be extracted using proposed methodology. Parametric estimation of the object radius and the estimation of the location parameters simultaneously is a challenging problem. Applying the application of processing steps on B-scan profiles incurs high computational costs, which is a limitation of the current methodologies. The metamodel itself is rendered using a novel deep-learning-based modified multilayer perceptron (M2LP) framework. The presented object characterization technique is favourably benchmarked against the state-of-the-art regression techniques, including Multilayer Perceptron (MLP), Support Vector Regression Machine (SVRM), and Convolutional Neural Network (CNN). The verification results demonstrate the average mean absolute error of 10 mm, and the average relative error of 8 percent, both corroborating the relevance of the proposed M2LP framework. In addition, the presented methodology provides a well-structured relation between the geophysical parameters of object and the extracted hyperbolic signatures. For the sake of supplementary verification under realistic scenarios, it is also applied for scenarios involving noisy data. The environmental and internal noise of the GPR system and their effect is analyzed as well. Furthermore, the proposed surrogate modeling approach is validated using measurement data, which is indicative of suitability of the approach to handle physical measurements as data sources.

## Introduction

Ground Penetrating Radar (GPR) has been widely used for underground investigations as a remote sensing tool that is based on electromagnetic wave theory^1–3^. In a rudimentary GPR system, time or frequency signals are transmitted and received via antennas that move along a path such as a synthetic aperture above the ground surface, and scan the underground. According to the requirements of the application, the antennas of different sizes, structures and frequency bands are employed such as a conventional monostatic C-Band horn antenna^4^, a transceiver X-Band cylindrical horn antenna^5^, a helical airborne GPR antenna with 480 MHz center frequency^6^. The movement of antenna systems along the scanning axis, the reflected received signal at one point renders the A-scan (1-D signal) data. During the scanning process, the collected A-scans are merged into B-scan images^7^ (2-D data). In the literature, most of the studies are based on investigating scattered fields from the buried object by using B-scan images, especially hyperbolic signatures and hyperbolic patterns^8–15^. A buried cylindrical target such as a rebar, a pipeline, and a wire (energy, optical, or signal cable) is subject to a hyperbolic regression in the B-scan^8^. A recognition of hyperbolic signature’s (pattern) geometrical features is the most common approach to detection, localization, and estimation of the object size using both analytical, numerical, and artificial intelligence (AI) methods^16–22^.

The identification and characterization of buried object requires some pre-processing operations to analyze only the reflected signals due to the object. One of them is correlation-based whitening algorithm^23^ applied on the scattering parameters to discriminate between reflections from air–soil boundary reflections and those associated with the buried object of from the buried object with different material types. In addition, the investigation of hyperbolic signatures derived from the B-scan data requires pre-processing operations. The most common operations include background subtraction and elimination of the air-ground surface echo; these are preferred to enable object recognition, and identification of the object-related properties such as localization and estimation of object size, material type, or shape^7–9,11,12,14–19,22,24,25^. In one of the studies^8^, following pre-processing, hyperbola extraction via Single Shot Multibox Detector (SSD)^8^ deep learning framework has been used to detect the objects and their localization. In an another study, to investigate of the hyperbola, a column-connection clustering (C3) algorithm^11^ has been proposed to determine the regions of interest. Subsequently, the neural network (NN) model has been used to classify C3 outputs for hyperbola identification. Also, an orthogonal-distance fitting algorithm^11^ has been applied to an identified hyperbola. A removal of subsurface reflection from the extracted hyperbola in the B-scan images (in the form of the amplitude and time vector) has been used to create inputs in a cascaded NN for characterization of the buried object^12^ with the help of the Hilbert transform (HT) to obtain enveloped signals. Another approach has been proposed for identification of a buried object and to obtain the reflected A-scans by using combined MD (Metal Detector) and GPR sensor^26^. The response features extracted from the peaks and their locations within the subsequent 1D time-varying amplitude signals by means of principle component analysis (PCA), are employed to classify the material type into three groups by using k-nearest neighbor supervised learning classification algorithm^26^. Other Artificial Intelligence (AI) algorithms have been successfully used for buried target recognition in GPR images include Deep Learning (DL), especially Convolutional Neural Network (CNN) frameworks^14–16,18,19,21^. 3D GPR data generated along longitudinal and cross axes is analyzed in CNN and LSTM (Long Short-Term Memory) units combined into a framework of a cascaded structure for the detection of buried explosive objects and discrimination targets or non-target alarms^15^. In a study of object detection^16^, CNN is used together with Long Short Term Memory (LSTM) network for the detection of a cylindrical object. In addition, nine different diameters of objects are classified in the extracted hyperbola regions within B-scan images^16^ generated using the gprMax toolbox^27,28^. The customized deep learning network as CNN framework is applied with SVM classifier instead of softmax layer^24^, and this structure is proposed for classification of B-scans generated by using gprMax simulation tool^27,28^ in terms of soil type, material type and object shape. Another approach is permittivity mapping of the subsurface structures for lining detection^29,30^ using customized CNN, and deep neural network frameworks. Using these tools, inversion of dielectric images can be obtained from the B-scan data. A similar approach has been applied to obtain permittivity inversion of geo-structures of buried targets by using deep neural network architectures^31^.

Detection is not the only problem considered in the context of buried object characterization. Some studies focused on investigating properties such as material type, material shape classification, localization, medium dielectric features, and object size estimation^12,22,32^ via AI surrogate models including cascading networks. In^22^, B-scan images generated by means of gprMax electromagnetic simulator toolbox^27,28^ have been pre-processed, and the images (along with the results of material type classification via Support Vector Machine (SVM), hyperbola curvature, and the object depth) have been used as inputs to obtain an estimation of object size using Gaussian Process Regression (GaPR)^22^. In another study^32^, a framework incorporating a Random Forest (RF) routine and NN regression model has been used to predict the object radius, depth, and the water content of the subsurface, based on the compressed reflected signal. The procedure is an exemplary study on the cascaded networks, with the regression parameters such as depth and water content being independent of each other, and the radius being dependent on the remaining parameters^32^. A-scan data samples have been employed as inputs for buried object characterization to obtain practical processing and reduction of the necessary computational resources for generating 2000 training samples^32,33^.

This work proposes a novel methodology for buried object characterization in a computationally-efficient approach via use of data-driven surrogate modelling. The ground reflections are removed, and background subtraction operations are executed so that B-scan image processing can be carried out. The surrogate is constructed at the level of hyperbolic patterns extracted using linear regression^34,35^ The latter results in dimensionality reduction of the parameter space^36^ and enables efficient analysis of GPR data using neural network models. Further, 2-D B-scan data is reduced to 1-D data consisting of electric field amplitude values along the scanning axis, so that an advantage is obtained with regard to computational cost of the proposed surrogate modeling approach. The estimated hyperbolic patterns contain information about the geophysical properties of the buried object, including its lateral position, depth, and radius. The underlying surrogate modelling technique involves deep-learning-based modified Multilayer Perceptron (M2LP) framework. Its architecture, in terms of deep-learning-based layers, is similar to a regression model employed to represent scattering parameters of a capacitively-fed antenna^37^. There are two fundamental contributions of this work. The first one is the employment of linear regression techniques for extracting hyperbolic signatures from the B-scan data, which are associated with the geophysical parameters of the buried object, and allow for dimensionality reduction of the dataset being handled in the process. The second is a novel deep- learning-based M2LP framework, which enables significant improvement of the computational efficiency of surrogate model rendition, thereby expediting the object characterization process.

The remaining part of the paper is arranged as follows. The next section formulates the buried object characterization task. It also provides a brief explanation of the GPR model, and the arrangement of the training samples. The subsequent section elaborates on a regression-based hyperbolic pattern extraction from the B-scans, as well as deep-learning-based modified MLP (M2LP) framework developed to perform characterization of depth, lateral position, and radius of the detected object. The following part extends our approach to handle noisy datasets, whereas the follow-up section also extends it to measurement data sets. The last section concludes the paper.

## Materials and method

This section formulates the buried object characterization task, as well as discusses the computational model of the ground penetrating radar (GPR) utilized in this context. Further, it provides detailed information about the data structures obtained from the GPR model and processed by the proposed object characterization algorithm.

### Problem formulation

**Figure 1:**
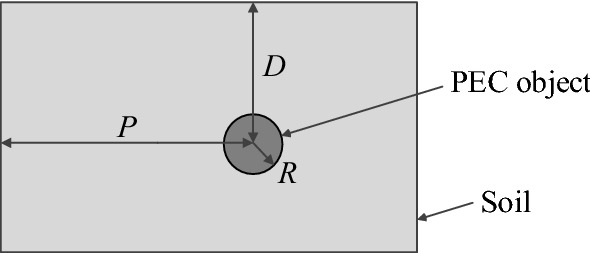
Buried object characterization problem: graphical illustration and definition of terms. Variables *R*, *D*, and *P* stand for the object radius, its depth, and lateral positions, respectively, all to be identified in the characterization process**.**

The problem at hand is to estimate characteristic parameters of a buried cylindrical object, specifically its depth *D*, lateral position *P*, and radius *R*. The meaning of these parameters has been explained in Fig. 1. The object itself is assumed to be made of perfect electrical conductor (PEC).

The estimation of the object parameters is realized here using fast surrogate model established using pre-processed data obtained from the GPR model to be discussed later. Data pre-processing allows for a considerable reduction of the dataset complexity, including a reduction of its dimensionality, thereby facilitating the modeling process. The latter is carried out using a dedicated M2LP framework elaborated on below.

### Configuration of GPR model

**Figure 2:**
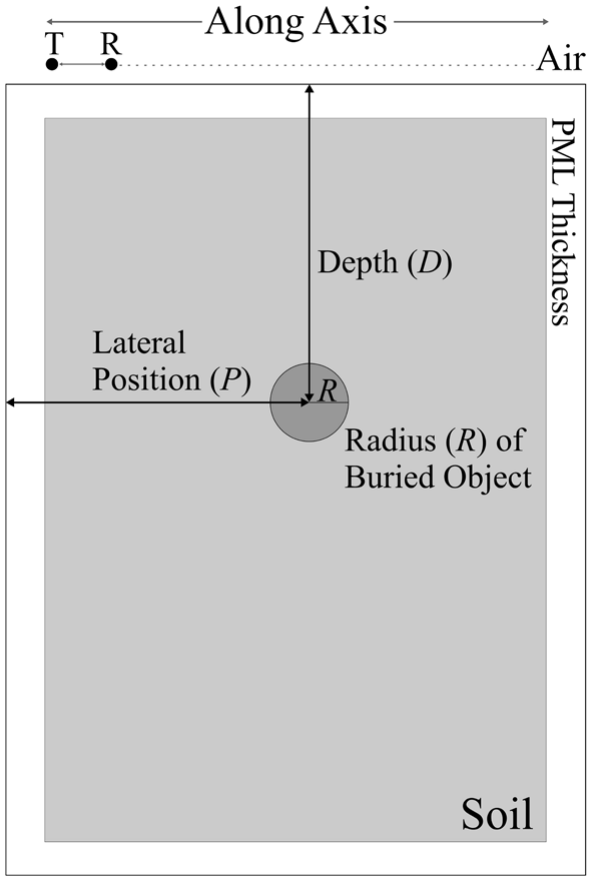
Configuration of the GPR model for generating training and testing data used by the proposed surrogate-assisted buried object characterization framework. T and R stand for the transmitter and receiver antenna, respectively. As mentioned earlier, the parameters to be estimated are the lateral position P, depth D, and object radius R.

In this work, the data used for object identification is obtained from the computational model representing the ground penetrating radar (GPR) together with the associated environment (a soil section containing the object buried therein). The GPR model is evaluated using the electromagnetic simulation software gprMax that involved Finite Difference Time Domain (FDTD) solver^27,28^. The geometrical configuration of the model has been presented in Fig. 2. As mentioned earlier, the object is represented as perfect electric conductor (PEC) in the form of a wire, pipe, or rebar. The travelling time *t*_*d*_ of the wave transmitted by the antenna can be computed as

1 $$t_{d} = \frac{{\sqrt {\varepsilon_{r} } 2d}}{c}$$

where *d* is the object depth, *ε*_*r*_ is the relative permittivity of the subsurface, and *c* is the speed of light in the free space. Note that the wave propagation time is monotonically dependent on the depth but also the subsurface permittivity.

The GPR model is configured as follows:

- The subsurface domain is defined as dry soil of relative permittivity 3 and conductivity 0.001 S/m;
- Subsurface domain dimensions are 0.4 m (lateral) and 0.6 m (vertical);
- The cubic cell size for the spatial discretization of the simulation environment is 1 mm;
- The boundary conditions are defined to be Perfectly Matched Layers (PML) with the thickness of ten cells (at all domain faces);
- The transmitter and receiver antennas (marked at *T* and *R* in Fig. 2, respectively), are placed 75 mm (almost 0.66*λ*^9,22,24^, *λ* being a guided wavelength) apart from each other and close to the ground surface (the distance is set to 2 mm^24^);
- The radius *R* of the buried object is assumed to be within the range 10 mm ≤ *R* ≤ 40 mm.

The GPR model is used to generate so-called A-scans^1–3,8,10^, which are waveforms of the electric field strength recorded by the receiver antenna over the 7.5 ns window. The antennas are moved for each trace (A-scan) at the along axis (scanning path) as shown in Fig. 2. The source signal *w*(*t*) is set to be a normalized first-order derivative of a Gaussian waveform with a center frequency *f*_*c*_ = 1.5 GHz. We have (the unit of time *t* are seconds)

2 $$w(t) = - 2\pi f_{c} \sqrt e \left( {t - \frac{1}{{f_{c} }}} \right)e^{{ - 2\pi^{2} f_{c}^{2} \left( {t - \frac{1}{{f_{c} }}} \right)^{2} }}$$

**Figure 3:**
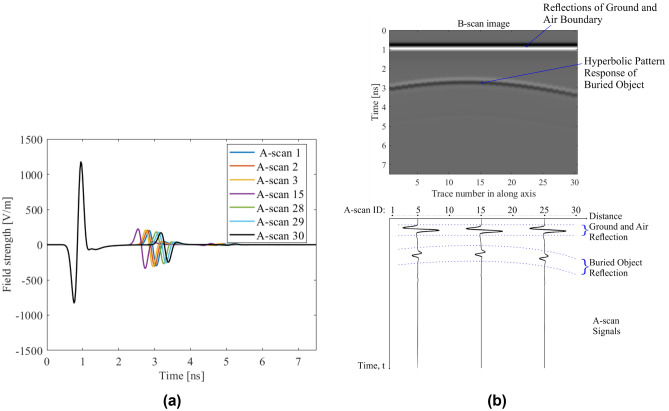
Signals obtained from the GPR model: (**a**) exemplary samples of raw A-scan signals from the test object set at D = 177 mm, P = 175 mm, and R = 19 mm; (**b**) B-scan image construction for the same test object.

Figure 3a shows exemplary A-scans obtained for a specific scenario. In addition, –3 dB level interval of the used waveform is approximately 1.1 to 2.7 GHz, and the approximate frequency bandwidth is 1.6 GHz, which is provided at the same time via the inverse of the pulse width gave bandwidth for the impulse radar system^1,2^. According to time window (*T* = 7.5 ns) of the one-point scanning process, frequency bin spacing (Δ*f*) at the bandwidth (*f*_BW_) is approximately as 133.4 MHz (1/*T*), the frequency step number, *N* is calculated 120 by using *f*_BW_/Δ*f*. The range resolution^1,2,10^ is also found as 54 mm by using the Fourier theory, which explains the relation between range resolution with velocity of the waveform in the soil medium as directly proportional to the range resolution and with frequency bandwidth as inversely proportional to the range resolution^10^. The maximum penetration depth^1,2,10^ for this configuration of the model is calculated as 0.649 m by using dielectric features of the subsurface, the value of frequency bin spacing, and the number of frequency steps. The maximum depth for object detection is related to the features of the excitation waveform and dielectric features of the surface. For example, in very lossy subsurface media, the amplitude of the signal is attenuated in a shorter time interval, so scanning operations may be limited to the shorter depth. When the objects are deeply buried, some techniques can be used such as “dewow” filtering^1,2,13^, band-pass filtering^2,38^ and time-dependent gain function in other words time-varying gain processing^1,2,38^.

**Figure 4:**
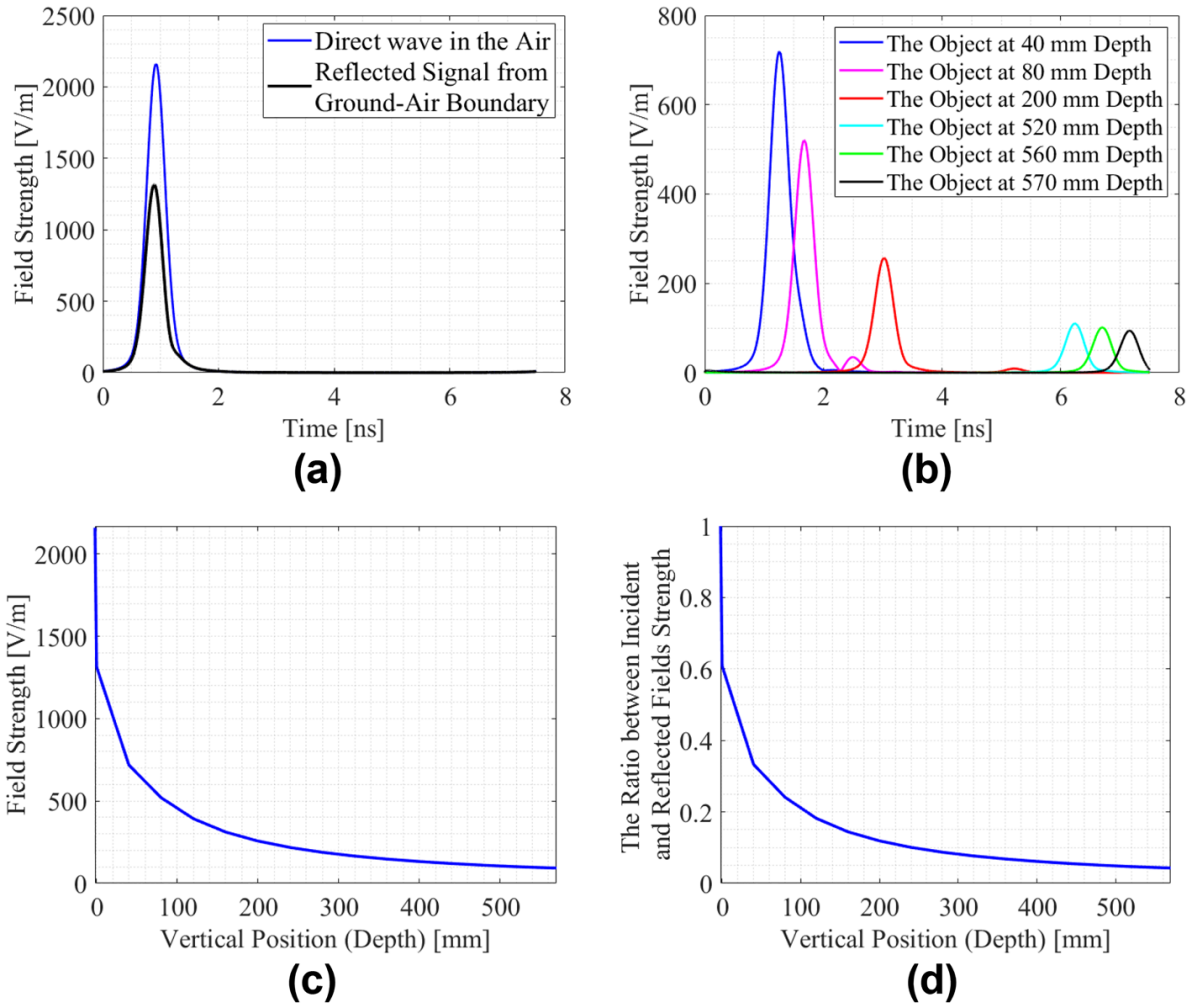
Reflected Signals from air-ground boundary and the objects in different depths with their enveloped forms: (**a**) the received direct wave in the air and reflection from the air-ground boundary; (**b**) sample scenarios of the A-scan to obtain soil attenuation along the penetration path with the object set at D = 40 mm, 80 mm 200 mm, 520 mm, 560 mm, 570 mm, and P = 238 mm as well as R = 10 mm; (**c**) amplitude change of the reflected signals versus the depth due to soil attenuation; (**d**) amplitude ratio between the incident signal and the reflected signals versus the depth.

While the excitation signal is propagating in a dielectric medium, its amplitude decreases depending on the dielectric features of the environment and the propagating path. With the defined parameters, attenuation coefficient^2^ is calculated as 0.1088 Np/m. In addition, the environment with the buried object in the center of antennas for lateral position and depths defined at intervals of 40 mm is simulated, and time-varying amplitudes are obtained. For these simulations, a cylindrical PEC object with the radius of 10 mm is used, which is buried at defined geometrical positions. In a particular simulation, the scenario is defined as 238 mm lateral position, 200 mm depth and 10 mm radius. Moreover, as mentioned for GPR model configuration, the antenna system is placed at a distance of 75 mm, and approximate value of 0.66 λ^9,22,24^ (wavelength of guided electromagnetic wave) and very close to ground upper surface. In other words, their distance from the ground is 2 mm^24^. Also, it differs from the conventional radar system due to the short target range and lossy propagation medium for electromagnetic waves^2^. In the proposed model, if there is no buried object in the subsurface, the transmitted field (of the incident field) from the air-ground boundary propagates until the maximum depth of defined surface domain, and when transmitted electromagnetic wave arrives the bottom of the subsurface domain, the amplitude will be zero due to the PML boundary conditions. For this reason, the soil attenuation and the propagation loss, due to the propagation in the dielectric subsurface medium, was demonstrated with the varying amplitudes of the reflected electric fields from the buried object at different depths. In Fig. 4a, a Hilbert transformed version of the received signal in the air without subsurface medium and reflected signal from air-boundary ground are represented. This representation is obtained from the Hilbert transform of the signals to utilize the enveloped form of the reflected electric fields. However, background subtracted raw signals are used in the modeling approach and transforming the enveloped form of the signals are used for the explanation of relation between the depth and amplitude change due to soil attenuation.

After the reflection from the upper ground surface, the transmitted part of the excitation signal propagates in the soil environment (subsurface domain), and it attenuates along the propagation path due to the soil dielectric features. In Fig. 4b, sample A-scans corresponding to the mentioned scenarios have been demonstrated to explain the relation between the depth and the magnitude of reflected electric field from the buried object. The principle of decreasing the amplitude of the reflected field as a function of increasing the depth at which the object is buried is used in this work to reduce dimensionality of data through linear regression, more specifically, by extracting the hyperbolic signature. This is the effect of soil attenuation on the amplitude of the reflected signals. In addition, the amplitude change of the reflected electric field due to the soil attenuation has been demonstrated in Fig. 4c by using buried object at different depths, reflection from air-ground boundary and direct wave in the air as well. The variation of amplitudes of the received signals caused by the buried object is explained by using the maximum (peak) amplitude of the enveloped signals. In addition, in Fig. 4d the ratio between incident and reflected electric fields coming from the buried object at different depths has been shown along with the varying amplitudes of the fields, which indicate the losses.

### Data structure: B-scan arrangement

The A-scans are parameterized using a lateral position of the T/R antenna system; *k* = 30 positions are considered altogether, as indicated in Fig. 3. At the same time, each A-scan is sampled at *N*_*t*_ = 3181 time steps. This data is aggregated into raw B-scans, in the form of an *N*_*t*_ × *k* matrix that contains the magnitude of the received electric fields strengths in V/m. Thus, the raw B-scan is represented as ***E*** = [*E*_*ij*_], with *i* = 1, …, 3181, and *j* = 1, …, 30 and ***E* = *E***_***target***_** + *E***_***clutter***_, ***E***_***target***_ includes reflections from the object and ***E***_***clutter***_ includes background reflection^7,25^. The background subtraction operation is applied to reconstruct 2D data (B-scan) as pre-processed B-scan that consists of just reflections from the target^25^. The initial pre-processing is then executed to reduce the data in the time domain by selecting only one out of ten time samples. This leads to compressed B-scans of the size 319 × 30. The process of constructing the B-scans has been illustrated in Fig. 3b.

The problem to be solved is to estimate characteristic parameters of a buried cylindrical object, particularly its depth *D*, lateral position *P*, and radius *R* by using computationally-efficient surrogate modeling approach. According to these characteristic parameters, scenarios are constituted and simulated to obtain B-scans. The pre-processing operation is applied to the collected B-scan data to eliminate the effects of background environment with the effects of other clutters commonly observed in data samples. It is important to elaborate on the structure of the A- and B-scans, as these are critical from the point of view of object characterization. Each A-scan presents two sets of ripples that correspond to the ground and air reflection (the first ripple) and the target reflection (the second ripple). The geophysical parameters of the object but also subsurface permittivity determine both the strength of the reflected field and its time allocation. Further, lateral relocation of the T/R antennas at the along axis leads to creation of a specific pattern of the reflected fields, referred to as hyperbolic signature (hyperbolic feature)^4,8,12,18,31,39^. The latter will be exploited in the proposed identification procedure as described in next section.

### Linear-regression-assisted surrogate modeling for buried object characterization

This sub-section outlines a linear regression technique employed to identify hyperbolic signatures of the B-scan data, as well as a novel deep-learning-based approach, Modified Multilayer Perceptron (M2LP) framework used to carry out buried object characterization. The following sub-sections provide the details of both techniques. The programming environment is Matlab.

### Hyperbolic signature extraction by linear regression

When the transmitting and receiving antenna system is moved along the scanning path, a specific pattern is produced as a result reflection from the buried object^4,8,12,18,31,39^. It is referred to as a hyperbolic signature, and encodes information about the significant characteristic parameters of the buried object. In this work, the hyperbolic signature is extracted using linear regression^34,35^.

The analytical form of the underlying regression model is a second-order polynomial, which provides a sufficient number of degrees of freedom to represent the object-related information in an adequate manner. The specific data encoded therein is amplitude of the reflected electric field versus the lateral position along the scanning path. A fundamental advantage of this sort of representation is simplification of the data structures being processed by the object characterization framework, both in terms of its sheer amount and dimensionality.

The hyperbolic signature extraction algorithm works as follows. In the first step, the amplitude maxima of each A-scan data acquired along the scanning path are identified for the entire wave travelling time period. The first maximum is the result of reflections from air and the ground upper surface for all A-scan signals, whereas the second maximum emerges from the target reflection. After eliminating the soil effect by applying background subtraction^7–9,11,12,14–19,22,24,25^, the estimated hyperbolic signature is created by gathering the pairs of second-highest maxima and the corresponding lateral locations. This data contains information about the buried object position (lateral position *P*, depth *D*), but also its radius *R*. In particular, when radius of the object is increased for at the same depth, the electric field amplitude is also increased. Also, the effects of reflection are observed for a larger number of consecutive A-scan signals around the position where the object is buried, as compared to the object featuring a smaller radius. In other words, the width of hyperbolic pattern increases.

In general form of relation between travelling time and the distance^8,18,20,40^ is given in Eq. (1) indicating two-way travel time. In detail, two-way travel time of the transmitting and receiving signals can be expressed by adding two different times. The reason is that the distance between the antennas and the object center are different from each other. The absolute distance to the object center depends on the distance between the antenna, horizontal and vertical distances between the antennas the object center, but also the object radius. In particular, the antennas are placed on close to the ground surface in the considered model, so that vertical distances of antennas to the object center are neglected.

**Figure 5:**
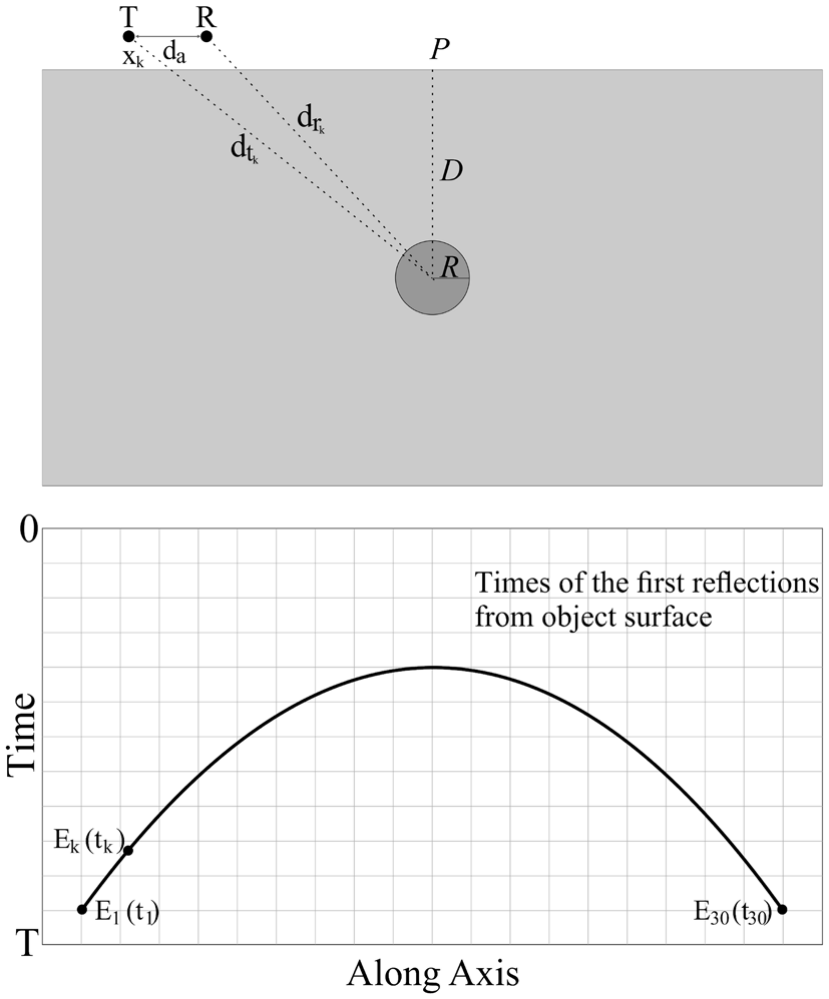
The parameters associated with the hyperbolic pattern constructed by the required time of the first reflections from the buried cylindrical object.

The specific pattern is constructed from reflections by using the arrival times of the first reflections from the buried object surface for each A-scan in the scanning path. Figure 5 shows the geometrical parameters associated with the hyperbolic pattern and its construction from the first reflections due to the buried cylindrical object according to the two-way travel time of the wave are demonstrated. The A-scan index is marked as *k* and ranges from 1 to 30. Mathematical modeling of the hyperbolic pattern and the times of the first reflections from the object surface is explained with the geometrical configuration and parameters^8,9,18,20,40^ of the data acquisition model. The distance *d*_*t*_ from the object center to the transmitter antenna, and *d*_*r*_ to the receiver antenna, as well as the distance from the transmitter antenna to the starting point at the scanning axis *x* for each A-scan is defined by calculating the hypotenuse

3 $$d_{{t_{k} }} = \left( {(x_{k} - P)^{2} + D^{2} } \right)^{1/2}$$

4 $$d_{{r_{k} }} = \left( {(x_{k} - d_{a} - P)^{2} + D^{2} } \right)^{1/2}$$

where *d*_*a*_ is the distance between the antennas (75 mm), *P* gives the lateral position of the center of object to the starting point, whereas *D* is the vertical distance between the center of the object and the upper ground surface. The required time *t* of two-way travelling wave for each A-scan of the first reflection is defined by

5 $$t_{k} = \sqrt {\varepsilon_{r} } \frac{{\left( {d_{{t_{k} }} + d_{{r_{k} }} } \right) - 2R}}{c}$$

where *ε*_*r*_ is the relative permittivity of soil medium and *c* is the light velocity in air. The hyperbolic signature is the merged form of reflected electric fields at time *t*_*k*_ for each A-scan as hyperbolic line and this data is described by

6 $$HP\left( k \right) = \left[ {\begin{array}{*{20}c} {E_{1} \left( {t_{1} } \right)} & {E_{2} \left( {t_{2} } \right)} & {E_{3} \left( {t_{3} } \right) \cdots \cdots \begin{array}{*{20}c} {E_{k - 1} \left( {t_{k - 1} } \right)} & {E_{k} \left( {t_{k} } \right)} \\ \end{array} } \\ \end{array} } \right]$$

The assumed analytical form of the function representing the aforementioned pattern is a second-order polynomial of the form

7 $$y(x) = ax^{2} + bx + c$$

where *x* is the lateral position (here, understood as the index of the A-scan), and *y* is the electric field strength corresponding to the second-highest maximum of the respective A-scan. The model is extracted based on the pairs {*x*_*k*_,*y*_*k*_}, *k* = 1, …, *N*, with *N* = 30. The least square regression problem is defined as

8 $$y_{k} = ax_{k}^{2} + bx_{k} + c\;\;{\text{for}} k = { 1}, \, ...,N$$

which can be written as

9 $$\mathop {\min }\limits_{[a,b,c]} \left\| {\left[ \begin{gathered} y_{1} \\ \vdots \\ y_{N} \\ \end{gathered} \right] - \left[ \begin{gathered} ax_{1}^{2} + bx_{1}^{{}} + c \\ \vdots \\ ax_{N}^{2} + bx_{N}^{{}} + c \\ \end{gathered} \right]} \right\|$$

or, in a matrix form

10 $$\mathop {\min }\limits_{[a,b,c]} \left\| {{\mathbf{y}} - {\mathbf{X}}\left[ \begin{gathered} a \\ b \\ c \\ \end{gathered} \right]} \right\|$$

where ***y*** = [*y*_1_ … *y*_*N*_]^*T*^, and

11 $${\mathbf{X}} = \left[ {\begin{array}{*{20}c} {x_{1}^{2} } & {x_{1} } & 1 \\ \vdots & \ddots & \vdots \\ {x_{N}^{2} } & {x_{N} } & 1 \\ \end{array} } \right]$$

The least-square solution to (11) is given as

12 $$\left[ \begin{gathered} a \hfill \\ b \hfill \\ c \hfill \\ \end{gathered} \right] = \left( {{\mathbf{X}}^{T} {\mathbf{X}}} \right)^{ - 1} {\mathbf{X}}^{T} {\mathbf{y}}$$

**Figure 6:**
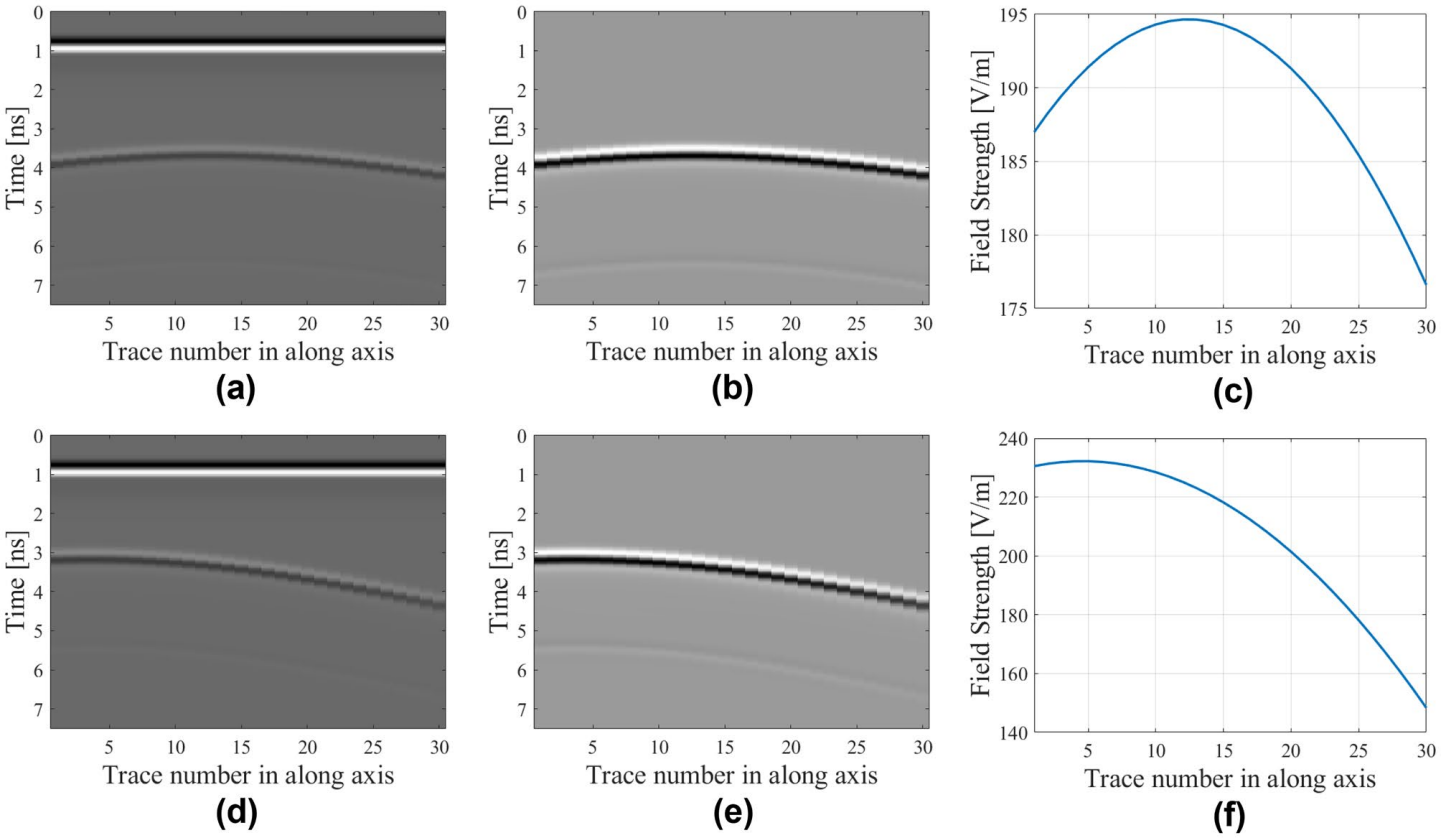
Raw and pre-processed B-scan data with their extracted hyperbolic patterns for selected samples concerning the following two test scenarios. The first scenario corresponds to *D* = 276 mm, *P* = 172 mm, and *R* = 33 mm: (**a**) raw B-scan image, (**b**) B-scan image with removed background reflections, (**c**) extracted hyperbolic pattern. The second scenario corresponds to *D* = 233 mm, *P* = 83 mm, and *R* = 34 mm: (**d**) raw B-scan image, (**e**) B-scan image with removed background reflections, (**f**) extracted hyperbolic pattern.

Figure 6 shows the examples of B-scan data along with their extracted hyperbolic patterns. Herein, a hyperbolic signature extraction is carried out using linear regression with the analytical form of the signature being a second-order polynomial. This methodology is also expressed via using linear regression function as activation function of the hyperbolic line *HP*(*k*) which is given by

13 $$y_{k} = \phi \left( {HP\left( k \right)} \right)$$

### Deep-learning-based modified multilayer perceptron (M2LP) for buried object characterization

**Figure 7:**
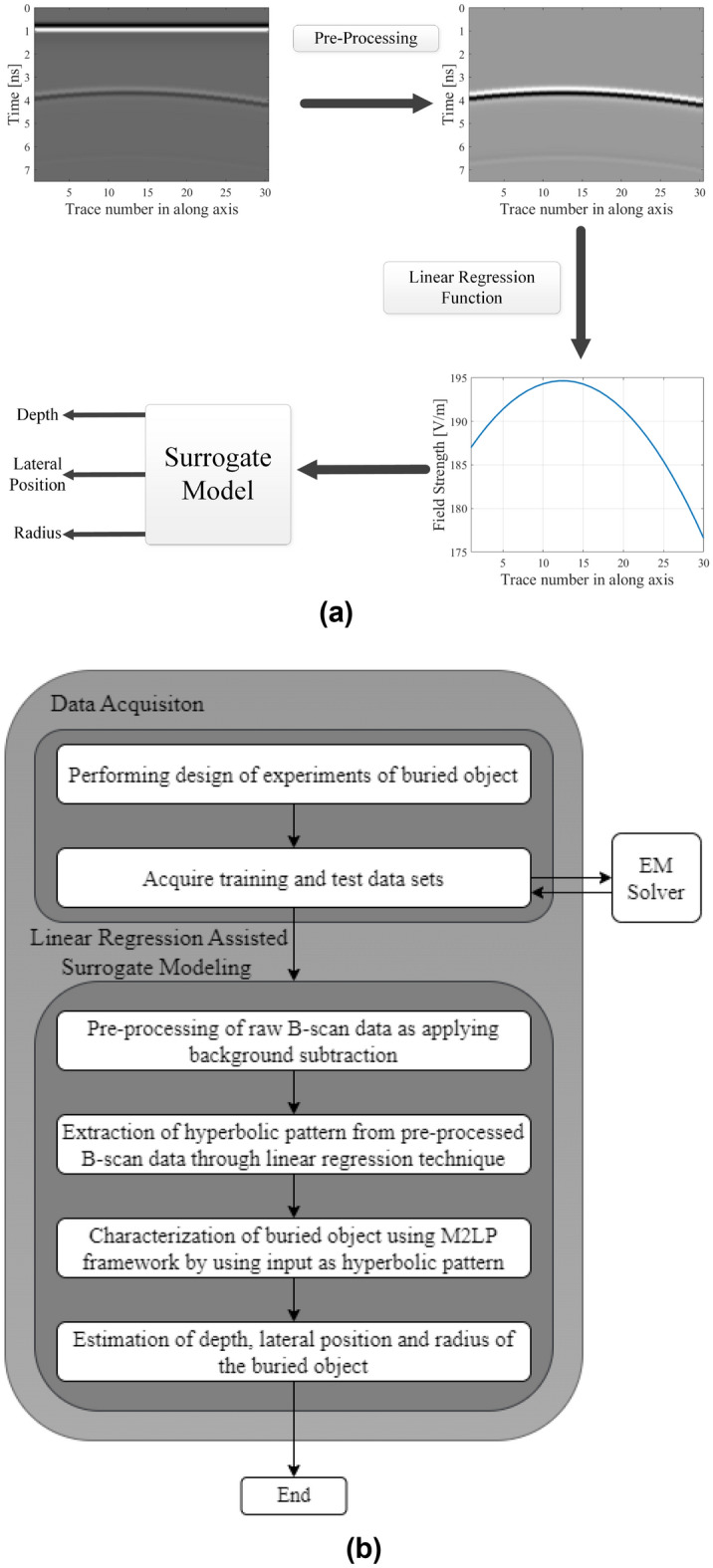
Modeling framework: (**a**) inputs and outputs of the phases in linear regression assisted surrogate modeling, (**b**) flowchart of the proposed data driven surrogate model for buried object characterization.

This sub-section introduces the proposed deep-learning-based modified multilayer perceptron (M2LP) model for buried object characterization. The flowchart of the modeling process as well as inputs and outputs of the phases of the proposed modeling approach for buried object characterization has been presented in Fig. 7.

The proposed framework belongs to a class of MLP networks as it contains fully connected layers. However, it also contains additional layers, normally employed in deep learning frameworks, including a batch normalization layer, Rectified Linear Unit (ReLU) as an activation function. Furthermore, the model is trained using the Adam algorithm^41^.

In contrast to the Convolutional Neural Network (CNN)-based regression models, the convolution and pooling layers are not included. In particular, data filtering leads to unused features remain in the data, also pieces of data losses as the hyperbolic patterns processed in our framework contain the attributes that already carry the knowledge about the characteristic parameters of the buried object. These can be used directly without further pre-processing.

**Figure 8:**
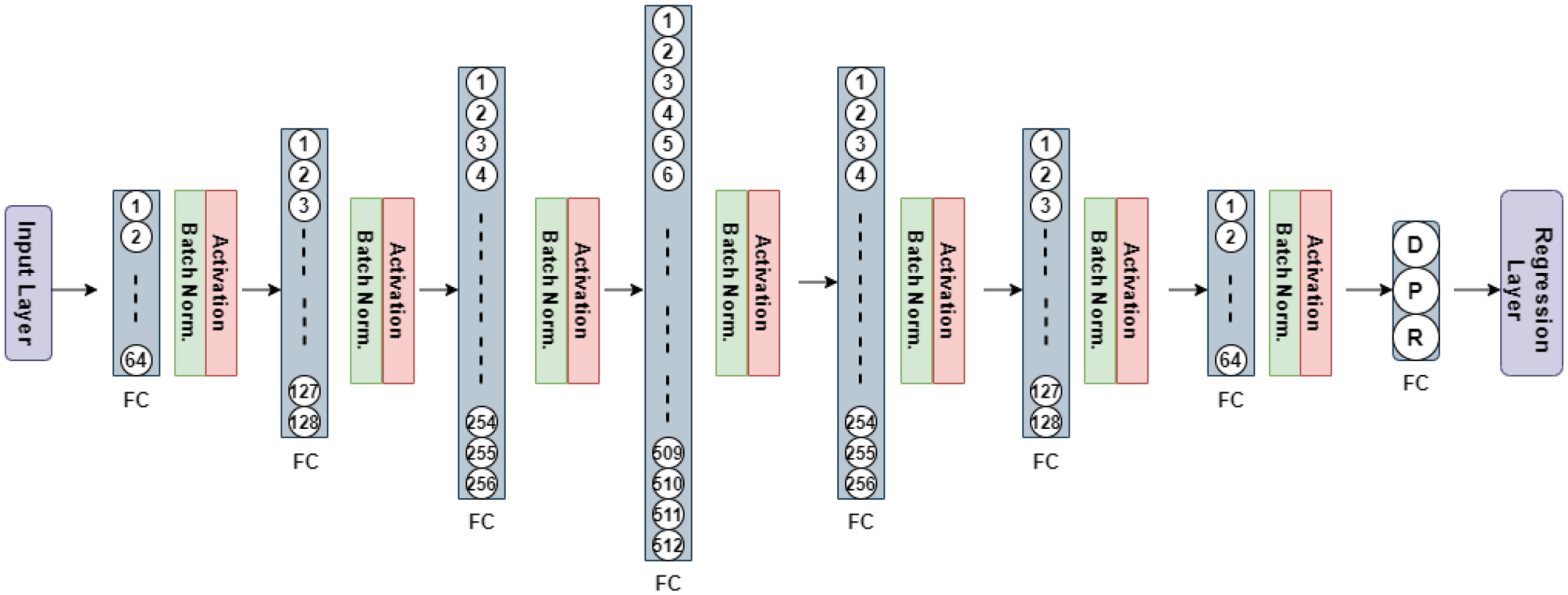
Architecture of the proposed M2LP framework.

The main architecture of the proposed framework has been shown in Fig. 8. It contains of a number of fully connected (FC) layers^37^, which operate similarly to feedforward neural networks. Moreover, each FC layer except the last one is followed by the batch normalization (BN)^42^ layer, incorporated to eliminate vanishing gradient issues. Further, the activation function employed here is ReLU, which is different from the sigmoid, logsig or tanh used in the traditional neural networks. The last FC layer consists of three neurons, which corresponding to characteristic parameters of the buried object to be estimated. The final layer is a regression layer producing the model outputs.

In the FC layer, neurons realize linear combinations of the input vector entries and adds bias, which is followed by executing the activation function (here, ReLU). The analytical form of ReLU is

14 $${\text{ReLU}} (x) = \left\{ {\begin{array}{*{20}c} x \\ 0 \\ \end{array} } \right. \, \begin{array}{*{20}c} {x \ge 0} \\ {otherwise} \\ \end{array}$$

**Figure 9:**
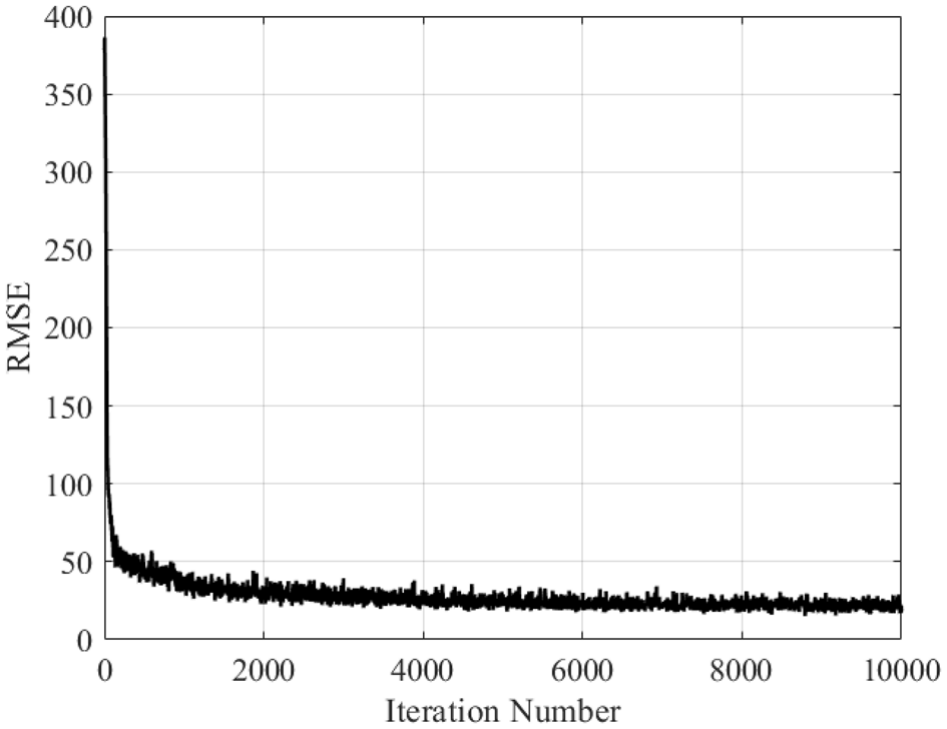
The training history of the proposed M2LP model in terms of RMSE (Root Mean Square Error) versus iteration number.

As mentioned earlier, the weights within the network are updated using the Adam^41^ algorithm, which is a variation of the back propagation method. Batch normalization^42^ technique is also utilized to standardize the layer inputs. It also stabilizes the learning process and dramatically reduces the number of epochs required to train the model. Herein, it is used with the batch size of one-tenth of the total number of the training data samples (e.g., 50 for the 500-sample set). The maximum epoch number is 1000, and the data is shuffled in each epoch. Figure 9 illustrates the training progress by providing the loss values versus the iteration number.

### Design of experiments

For demonstration and verification purposes, the proposed surrogate model has been constructed using the training data sets of different sizes, as explained in Table 1. Each sample corresponds to a different scenario concerning the buried object location (lateral position *P* and depth *D*), as well as its radius *R*. Both the training and testing datasets are allocated using Latin Hypercube Sampling (LHS)^43^.

**Table 1:** The arrangement of the training/testing data for the considered GPR problem.

Data sets	Total sample of scenarios
Training data set 1 (DS1)	100
Training data set 2 (DS2)	200
Training data set 3 (DS3)	300
Training data set 4 (DS4)	500
Training data set 5 (DS5)	800
Testing data set	50
The dimensions of the B-scans : 319 × 30	

### Benchmarking

In this section, the proposed surrogate modelling approach is compared to the state-of-the-art techniques utilized for buried object characterization. The benchmark methods include CNN^14,15,31,39^, MLP^3,11,13^, and SVRM^44^. Also, two of the benchmark cases are analyzed using the M2LP framework operating on different data sets generated by PCA. These methods and cases are briefly explained below.

CNN (Convolutional Neural Network) is a version of deep learning model^8,14,15,18,31^. The convolutional layer, which is one of the main components of the network, has the ability to automatically extract the data features owing to the convolution filter in the layer. In CNN, several blocks are utilized together, such as a convolution layer (filter), a batch normalization layer, a pooling layer, the activation function, and a fully connected (FC) layer, all involved in the hidden layer. The architecture and the hyper-parameter configuration of the CNN used here is as follows: three convolution layers followed by the batch normalization layer, activation function as ReLU (Rectified Linear Unit) layer, as well as the two pooling layers included after the last convolution layer, as well as a fully connected layer with three neurons to represent the system outputs. Other user-defined parameters, such as the size and the number of the convolution filter (32, 64, 128) and pooling layer are assigned according to literature recommendations^15^. In addition, it should be mentioned that—in the cited works—the input data is two-dimensional, consequently, the filters of the CNN layers are of the corresponding dimensionality, whereas the filters consisted of the CNN model used for benchmarking in this work are one-dimensional. The CNN model is trained using the Adam algorithm and a batch size of 50. The learning rate has been set to 10^–3^ until maximum epoch number reached to 1000.

Another benchmarking technique is MLP^3,11–13,45^. The model utilized here features the following hyper-parameter configuration: two hidden layers with 32 and 64 hidden neurons, respectively; log-sigmoid activation functions, training by the Levenberg–Marquardt algorithm until the maximum epoch number reaches 1000.

The last benchmark model is Support Vector Regression Machine (SVRM), which belongs to the class of supervised statistical learning techniques^9,13,44,46^. Herein, SVRM has been applied using Bayesian optimization for hyper-parameter adjustment. One of the important components SVRM is a kernel function. Here, it is selected as a Gaussian function to realize nonlinear mapping between the hyperbolic patterns and characteristic parameters of the buried object.

Table 4 gathers the performance metrics as well as the training time of the proposed and the benchmark methods. The breakdown of the modelling error for specific characteristic parameters of object (*P*, *D*, *R*) can be found in Table 5, whereas Table 6 provides prediction performance in concern with comparison of true and predicted geophysical parameters for selected scenarios. It can be observed that the predictive power of the proposed approach significantly better than for the benchmark methods. Both in terms of MAE and RME, the accuracy of our framework is about twice as good as the best benchmark technique (CNN), and about three times better than the accuracy of the remaining methods (MLP and SVRM). Figure 12 provides examples of specific scenarios and corresponding object parameter prediction for the proposed approach and the benchmark methods.

**Figure 10:**
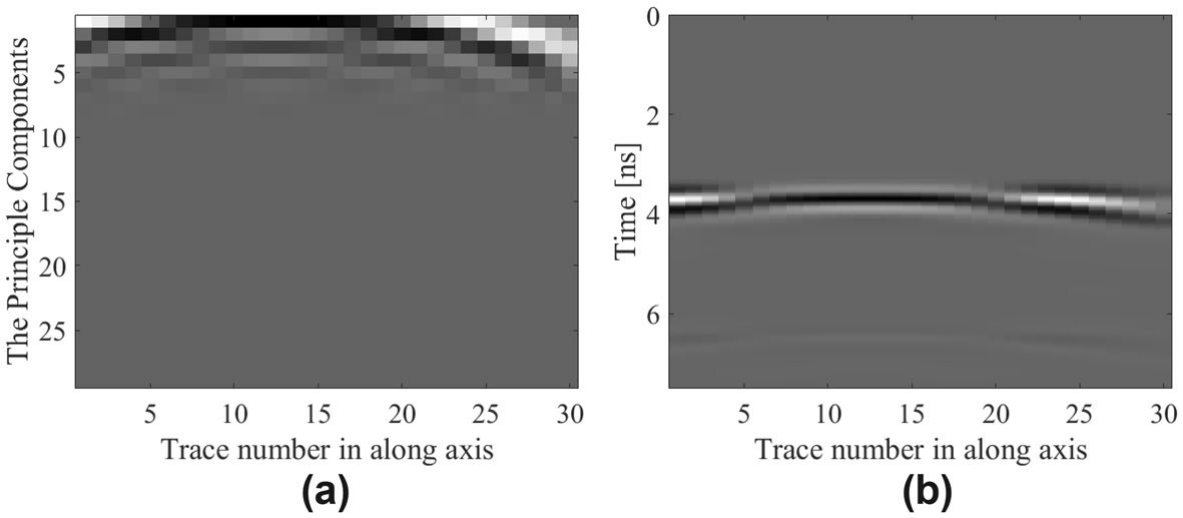
A sample test scenario corresponding to *D* = 276 mm, *P* = 172 mm, and *R* = 33 mm: (**a**) extracted principle component features by using PCA; (**b**) pre-processed (clutter reduced) B-scan image by using PCA.

Two of the benchmark cases include different data sets generated using PCA^26,47,48^. The first one consists of features extracted using PCA similar as in study^26^ for the purpose of dimensionality reduction of the B-scan. It is mentioned that B-scan data has the size of 319 × 30 (319 time steps and 30 A-scans). After applying PCA to raw B-scan, the array of principal components is obtained as the size of 30 × 29. Its rows are the A-scans and the columns are the components. The principal component array is transformed to 1D feature vector and its size for each B-scan 1 × 870. In addition, the vertical and horizontal coordinates of the mean and variance magnitudes are extracted from the peak amplitudes and depth indices array. As a result, the input vector is prepared to have the size of 1 × 874 for the proposed M2LP framework. The second benchmark case is specialized on clutter reduction by using PCA^47,48^, where only the reflections coming from the buried object are obtained and linear regression technique is employed to identify hyperbolic signatures of the B-scan data. After that, the proposed deep-learning-based approach, M2LP framework is used to carry out buried object characterization. In Fig. 10, for a sample test scenario the principal components as an image (in 2D data form), and a B-scan image pre-processed (clutter reduced) using PCA are demonstrated. In addition, the last benchmark case including a study of characterization of geophysical parameters with A-scan analysis^49^. The performance results of that study which is computationally efficient surrogate modeling via a novel deep learning-based framework that focuses on the object characterization in terms of its geophysical parameters with A-scan analysis^49^ and by using raw data (without any background subtraction operations) are added. These benchmark cases only have approximate training and testing time durations, so this data is not considered in the comparative study. In Table 7, performance metrics of the proposed and the benchmarking cases, in the form of the average values and standard deviations of MAE and RME over ten independent runs have been represented.

## Experimental results and discussion

The predictive power of the surrogate model (both the proposed one and the benchmark) is quantified using the Mean Absolute Error (MAE) and the Relative Mean Error (RME) defined as

15 $$MAE = \frac{1}{N} \times \sum\limits_{i = 1}^{N} {\left| {T_{i} - P_{i} } \right|}$$

16 $$RME = \frac{1}{N} \times \sum\limits_{i = 1}^{N} {\frac{{\left| {T_{i} - P_{i} } \right|}}{{\left| {T_{i} } \right|}}}$$

where *N* is the number of testing samples, whereas *T*_*i*_ and *P*_*i*_ are the target and model-predicted values, respectively, for the *i*th sample.

Table 2 shows the MAE and RME for all considered data sets and each characteristic parameter of the object, as well as the average error levels. It can be observed that the accuracy of the proposed framework is satisfactory for practical purposes already with the 500-sample set (DS4), for which the average MAE error falls below ten percent. Consequently, benchmarking of the model will be conducted using this particular dataset.

**Table 2:** Prediction performance of considered characteristic parameters for the proposed M2LP framework.

Data set	Characteristic	MAE [mm]	RME [%]	Average MAE [mm]
DS1 (100 samples)	Depth	43.9	13.3	27.5
	Lateral position	30.7	22.2	
	Radius	7.8	39.4	
DS2 (200 samples)	Depth	41.3	11.8	25.1
	Lateral position	27.4	16.8	
	Radius	6.5	26.2	
DS3 (300 samples)	Depth	22.6	6.4	13.5
	Lateral position	14.1	9.8	
	Radius	3.7	15.8	
DS4 (500 samples)	Depth	14.2	4.4	9.6
	Lateral position	11.8	7.3	
	Radius	2.6	10.8	
DS5 (800 samples)	Depth	11.6	3.7	7.6
	Lateral position	9.0	6.4	
	Radius	2.1	8.7	

**Figure 11:**
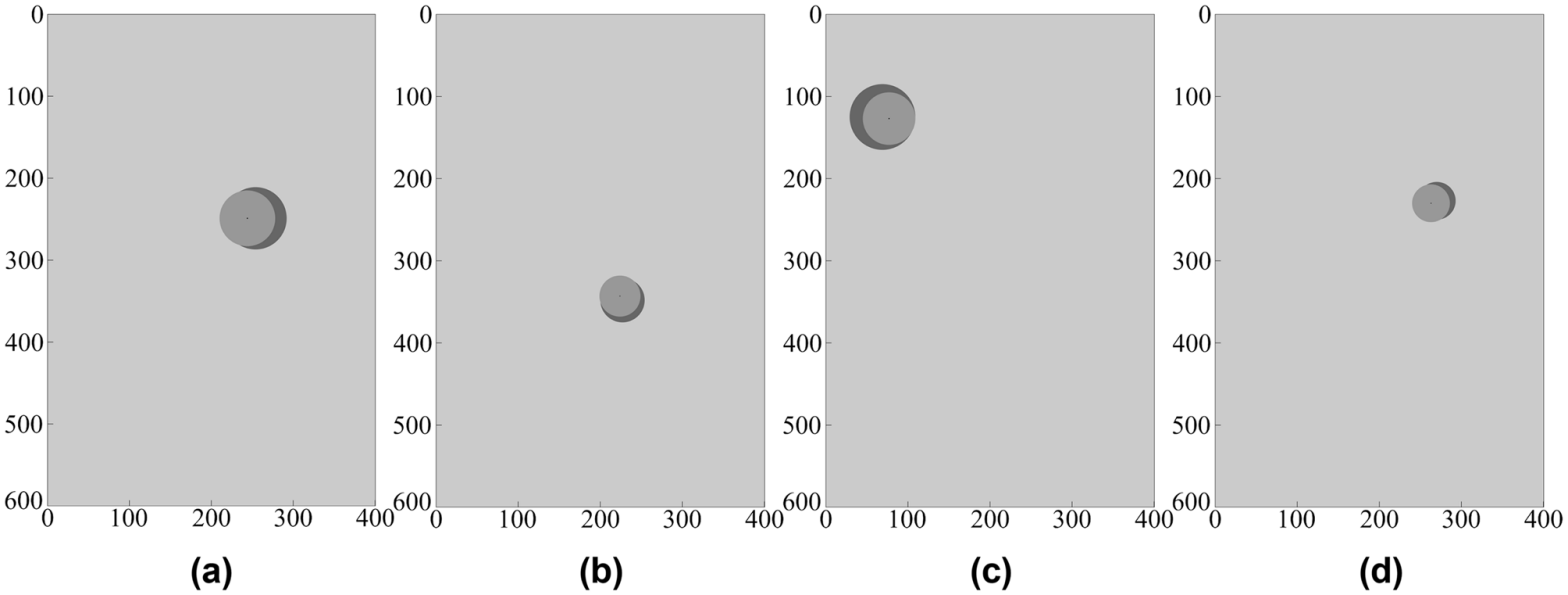
Prediction of geophysical parameters of the object, obtained using the proposed surrogate model (built based on the DS4 dataset). The target and surrogate-predicted object marked using the dark- and light-grey shade, respectively: (**a**) *D* = 249 mm, *P* = 254 mm, *R* = 38 mm, (**b**) *D* = 348 mm, *P* = 227 mm, *R* = 27 mm, (**c**) *D* = 125 mm, *P* = 69 mm, *R* = 40 mm, (**d**) *D* = 227 mm, *P* = 270 mm, *R* = 23 mm.

**Figure 12:**
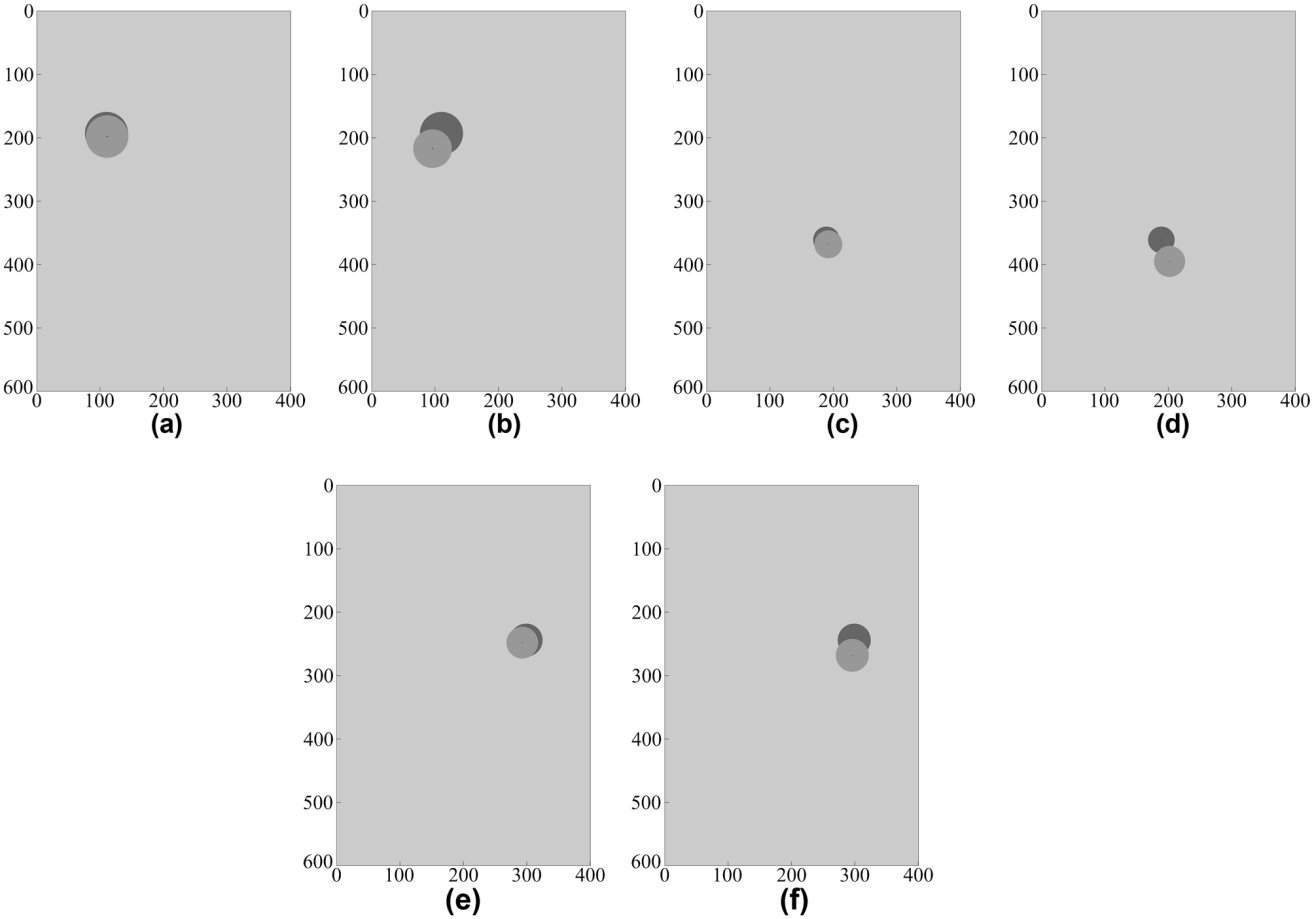
Prediction of geophysical parameters of the object, obtained using the proposed surrogate model and the benchmark techniques (all using the DS4 dataset). The target and surrogate-predicted object marked using the dark- and light-grey shade, respectively: Scenario I: *D* = 193 mm, *P* = 110 mm, *R* = 34 mm, (**a**) proposed model, (**b**) CNN; Scenario II: *D* = 361 mm, *P* = 189 mm, *R* = 21 mm, (**c**) proposed model, (**d**) MLP; Scenario III: *D* = 244 mm, *P* = 299 mm, *R* = 26 mm, (**e**) proposed model, (**f**) SVRM.

Figures 11, 12 illustrates some of the geometrical configurations in terms of alignment between surrogate-predicted and target parameters of the object. Table 3 provides numerical data, i.e., the target and surrogate-predicted object parameters for selected scenarios. As it can be observed, visual agreement between the actual and predicted object size and location is excellent (Tables 4, 5, 6, 7).

**Table 3:** M2LP-based estimation of the characteristic parameters in comparison with their true values for selected test scenarios.

	*D*	*P*	*R*
True value	152	102	32
M2LP [this work]	160	101	31
Error	− 8	1	1
True value	187	152	39
M2LP [this work]	187	152	33
Error	0	0	6
True value	198	146	14
M2LP [this work]	204	143	17
Error	− 6	3	− 3
True value	212	318	19
M2LP [this work]	214	311	20
Error	− 2	7	− 1
True value	217	201	29
M2LP [this work]	218	198	27
Error	− 1	3	2

Explanation of terms: *D*—depth, *P*—lateral position, *R*—radius (all in mm).

**Table 4:** Performance metrics and the training time of the proposed and the benchmark models, averaged with standard deviation over ten independent runs.

Model	MAE [mm]	RME [%]	Training time [min]	Processing time for a single input test data [ms]
CNN	19.8 ± 1.8	15.4 ± 1.7	6 ± 0.6	10 ± 0.4
MLP	29.6 ± 6.0	26.5 ± 3.4	19 ± 1.0	3 ± 0.2
SVRM	28.8 ± 5.3	24.2 ± 5.0	2 ± 0.3	2 ± 0.2
M2LP [this work]	10.4 ± 1.2	8.1 ± 0.9	4 ± 0.5	7 ± 0.3

The models trained using the dataset DS4 (500 samples).

**Table 5:** Breakdown of the best prediction performance of the proposed and the benchmark models, over ten independent runs.

Model	Characteristic	MAE [mm]	RME [%]	Average MAE [mm]
CNN	Depth	25.5	7.9	18.3
	Lateral position	22.6	16.3	
	Radius	6.6	26.5	
MLP	Depth	19.0	7.3	26.0
	Lateral position	51.5	35.0	
	Radius	7.3	36.5	
SVRM	Depth	39.7	15.7	23.7
	Lateral position	23.3	17.5	
	Radius	6.2	30.4	
M2LP [this work]	Depth	14.2	4.4	9.6
	Lateral position	11.8	7.3	
	Radius	2.6	10.8	

The models trained using the dataset DS4 (500 samples).

**Table 6:** Characteristic parameter prediction for the proposed and benchmark methods for selected test scenarios.

Model	*D*	*P*	*R*	Error (*D, P, R*) [mm]
True value	107	210	33	–
M2LP [this work]	111	208	30	− 4, 2, 3
CNN	121	201	24	− 14, 9, 9
MLP	113	212	21	− 6, − 2, 12
SVRM	125	202	32	− 18, 8, 1
True value	279	134	37	–
M2LP [this work]	277	130	36	2, 4, 1
CNN	275	132	30	4, 2, 7
MLP	249	191	26	30, − 57, 11
SVRM	248	132	27	31, 2, 10
True value	170	120	11	–
M2LP [this work]	177	117	14	− 7, 3, − 3
CNN	186	108	9	− 16, 12, 2
MLP	168	135	26	2, − 15, − 15
SVRM	287	154	25	− 117, − 34, − 14
True value	287	142	31	–
M2LP [this work]	287	147	31	0, − 5, 0
CNN	282	143	26	5, − 1, 5
MLP	267	194	26	20, − 52, 5
SVRM	282	150	26	5, − 8, 5
True value	260	187	15	–
M2LP [this work]	260	187	16	0, 0, − 1
CNN	258	194	13	2, − 7, 2
MLP	262	193	26	− 2, − 6, − 11
SVRM	334	169	24	− 74, 18, − 9

**Table 7:** Average performance metrics of the proposed and the benchmark cases and standard deviation, computed over ten independent runs.

Model and methodology	MAE [mm]	RME [%]
M2LP [this work]	10.4 ± 1.2	8.1 ± 0.9
M2LP with hyperbolic signature, reconstruction B-scan via PCA	17.4 ± 0.4	20.4 ± 0.4
M2LP with extracted features, the principal components and mean, variance coordinates	17.2 ± 0.9	21.3 ± 0.9
TFRM with unprocessed raw 1D data, A-scan analysis^49^	12.1 ± 0.1	16.5 ± 0.5

The results indicate that linear regression assisted hyperbolic signature approach with the proposed deep-learning-based M2LP framework features smaller error as compared to other cases including different data sets with the proposed framework and different method (A-scan analysis) with TFRM framework^49^. It can be observed that, in a qualitative sense, according to the presented results the proposed methodology is superior to the benchmark cases. As mentioned in the section on configuration of the GPR model, the scanning subsurface dimensions are 400 mm × 600 mm, and the maximum radius of the object is assumed to be 40 mm. The minimum distance between the object and the upper and bottom surface of the soil is defined as 60 mm. With respect to this comment, the effect of reducing the distance to the ground surface is analyzed by adding of a small group of scenarios (20 samples) that correspond to the object-to-surface distance of 20 mm and 30 mm. The depth characteristic parameter is defined as the distance between the ground surface and the center of the object, so the depth value changes according to the radius value of the object in the scenario. The generated B-scan data for this analysis also can be expressed as ***E*** = [*E*_*ij*_], with *i* = 1, …, 319, and *j* = 1, …, 30 and ***E* = *E***_***target***_** + *E***_***clutter***_, ***E***_***target***_ includes reflections from the buried object which is so close to the ground surface and ***E***_***clutter***_ includes background reflections^7,25^. The proposed methodology is followed for the new data set to analyze the effects of the object distance to the ground surface. Firstly, the pre-processing is executed by selecting only one out of ten time samples of the data in the time domain. This leads to compressed B-scans of the size 319 × 30. Another phase is applied as the background subtraction operation. The next step is hyperbolic extraction from the pre-processed 2D data. Further, 2-D B-scan data is reduced to 1-D data consisting of electric field amplitude values along the scanning axis. Finally, the proposed M2LP framework is used for buried object characterization and the results are demonstrated in Table 8.

**Table 8:** Characteristic parameter prediction of the proposed M2LP framework assuming short distance between the object and the ground surface.

Scenarios and error metrics	*D*	*P*	*R*	Closeness to the ground surface
True value	60	159	40	20
M2LP [this work]	54	159	35	19
Error [mm]	6	0	5	1
Absolute relative error [%]	10.0	0.0	17.5	5.0
True value	68	254	38	30
M2LP [this work]	62	260	34	28
Error [mm]	6	− 6	4	2
Absolute relative error [%]	8.8	2.4	10.5	6.7
True Value	43	270	23	20
M2LP [this work]	53	277	29	24
Error [mm]	− 10	− 7	− 6	− 4
Absolute relative error [%]	23.3	2.6	26.1	20.0
True value	50	344	30	20
M2LP [this work]	52	328	26	26
Error [mm]	− 2	16	4	− 6
Absolute relative error [%]	4.0	4.7	13.4	30.0

Explanation of terms: *D*—depth, *P*—lateral position, *R*—radius and closeness to the ground surface (all in mm).

### Data-driven surrogate modeling under realistic scenarios including noisy data

**Figure 13:**
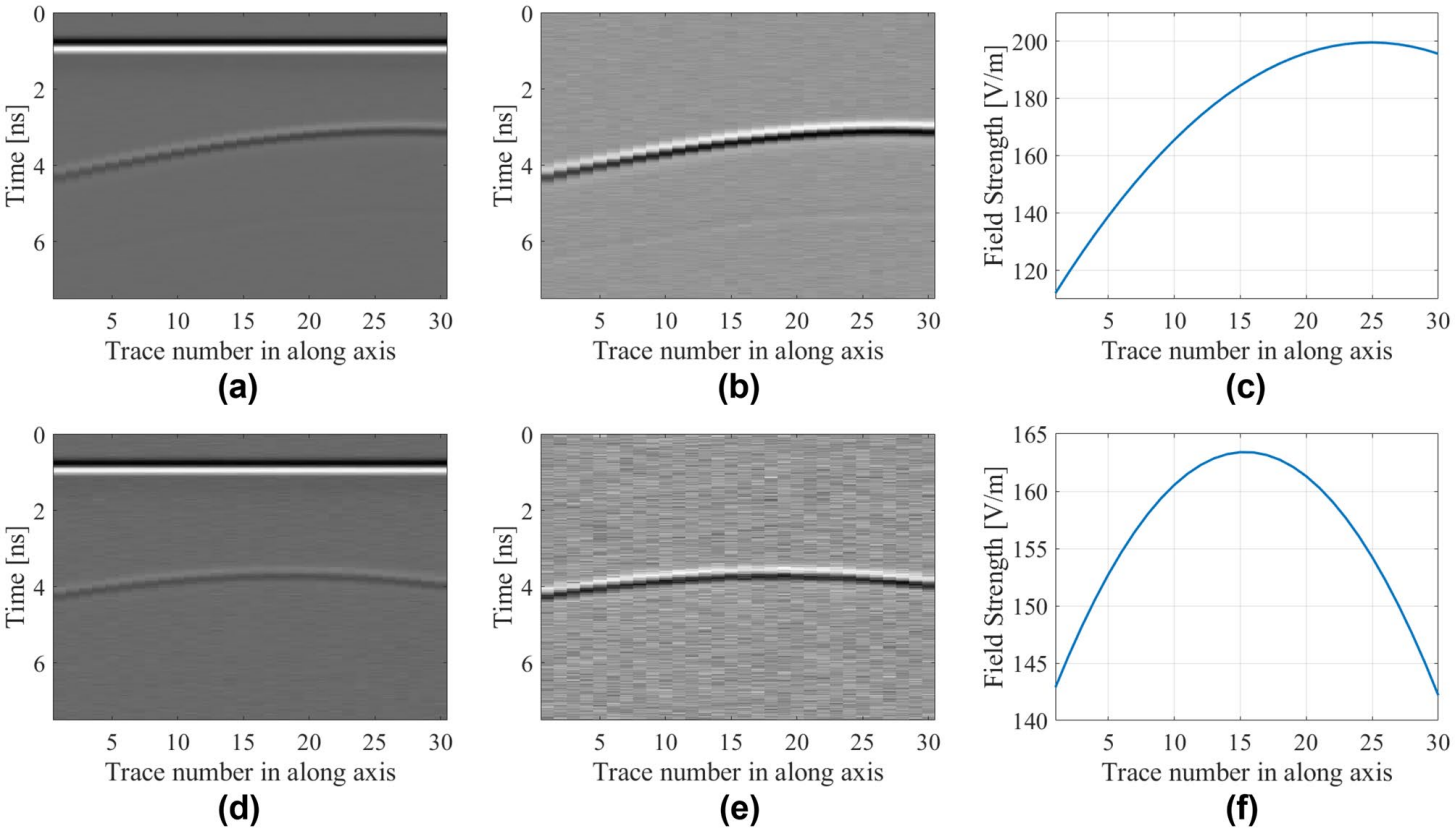
Raw and pre-processed B-scan data along with their extracted hyperbolic patterns for selected samples from the test scenarios including noise. The SNR for first scenario is 30 dB, whereas the objective parameters are *D* = 212 mm, *P* = 318 mm, and *R* = 19 mm: (**a**) raw B-scan image, (**b**) B-scan image with removed background reflections, (**c**) extracted hyperbolic pattern. The SNR for the second scenario is 20 dB, *D* = 259 mm, *P* = 231 mm, and *R* = 12 mm: (**d**) raw B-scan image, (**e**) B-scan image with removed background reflections, (**f**) extracted hyperbolic pattern.

This section addresses object characterization assuming more realistic scenarios, namely, noisy data. For demonstration purposes, new data sets were created by adding random noise to the A-scan samples from the DS4 dataset. This is to emulate the environmental and internal noise of the data gathering system in GPR the model, and to evaluate its effects on the proposed modeling methodology. The selected scenarios from the testing data have been shown in Fig. 13.

The literature offers different approaches to noise incorporation^18,29,30,39^. The background noise of real data has been integrated with the buried object reflections (B-scans) of synthetic data^18,29^. In a study^39^, a noisy data set has been generated by adding randomly replaced black and white pixels on B-scans. This allows for investigating different noise levels in synthetic data generated using the gprMax simulation tool to predict diameter of the target on the extracted hyperbola from the pre-processed B-scans^39^. In this work, the supplementary noisy data sets were created with different signal-to-noise (SNR) levels of 20 dB and 30 dB, by adding the white Gaussian noise^30^ to the data set including 500 samples (dataset DS4). This enables emulating conditions that are closer to the realistic situation or on-site applications.

For this investigation, the proposed M2LP framework has been compared with all benchmark models (CNN, MLP, SVRM). The results are presented in Table 9. Table 10 provides a comparison of model-predicted versus actual characteristic parameters for selected test cases. The results indicate that the proposed M2LP framework outperforms the benchmark by a considerable margin. In particular, the average MAE of M2LP is lower by a multiplicative factor of 1.6, 1.9 and 1.9 as compared to CNN, MLP, and SVRM, respectively for SNR = 30 dB, whereas the improvement is as high as 1.5, 1.8, and 1.6 over CNN, MLP, and SVRM, respectively, for SNR = 20 dB.

**Table 9:** Prediction accuracy of characteristic parameters of the buried object for all models (dataset DS4 with 500 samples).

Model	Characteristic	SNR = 30 dB			SNR = 20 dB		
		MAE [mm]	RME [%]	Average MAE [mm]	MAE [mm]	RME [%]	Average MAE [mm]
CNN	Depth	38.1	14.1	28.9	58.1	19.7	37.9
	Lateral position	43.0	27.6		48.9	30.8	
	Radius	5.5	25.2		6.7	31.4	
MLP	Depth	53.7	18.2	35.6	72.9	27.5	45.6
	Lateral position	45.8	30.0		56.6	37.2	
	Radius	7.1	36.3		7.4	36.6	
SVRM	Depth	47.8	18.9	35.8	61.5	24.1	41.1
	Lateral position	54.3	37.0		56.5	37.1	
	Radius	4.6	21.6		5.4	26.8	
M2LP [this work]	Depth	25.3	7.8	18.8	28.4	9.6	24.5
	Lateral position	26.6	17.6		39.3	24.5	
	Radius	4.6	21.2		5.8	27.7	

Considered scenarios include noisy data with SNR of 30 dB and 20 dB.

**Table 10:** Predicted characteristic parameters of the buried object versus true values for selected test scenarios.

Model	*D*	*P*	*R*	Error (*D, P, R*) [mm]
True value	170	120	11	–
M2LP [30 dB]	182	124	11	− 12, − 4, 0
CNN [30 dB]	196	114	17	− 26, 6, − 6
MLP [30 dB]	195	102	27	− 25, 18, − 16
SVRM [30 dB]	289	158	18	− 119, − 38, − 7
True value	372	196	35	–
M2LP [30 dB]	361	195	33	11, 1, 2
CNN [30 dB]	302	193	29	70, 3, 6
MLP [30 dB]	380	160	25	− 8, 36, 10
SVRM [30 dB]	323	199	28	49, − 3, 7
True value	276	172	33	–
M2LP [20 dB]	265	161	28	11, 11, 5
CNN [20 dB]	209	180	20	67, − 8, 13
MLP [20 dB]	307	200	24	− 31, − 28, 9
SVRM [20 dB]	288	189	28	− 12, − 17, 5
True value	143	333	25	–
M2LP [20 dB]	150	325	22	− 7, 8, 3
CNN [20 dB]	201	216	23	− 58, 117, 2
MLP [20 dB]	130	316	28	13, 17, − 3
SVRM [20 dB]	247	274	27	− 104, 59, − 2

### Data-driven surrogate modeling and object characterization with measurement data

**Figure 14:**
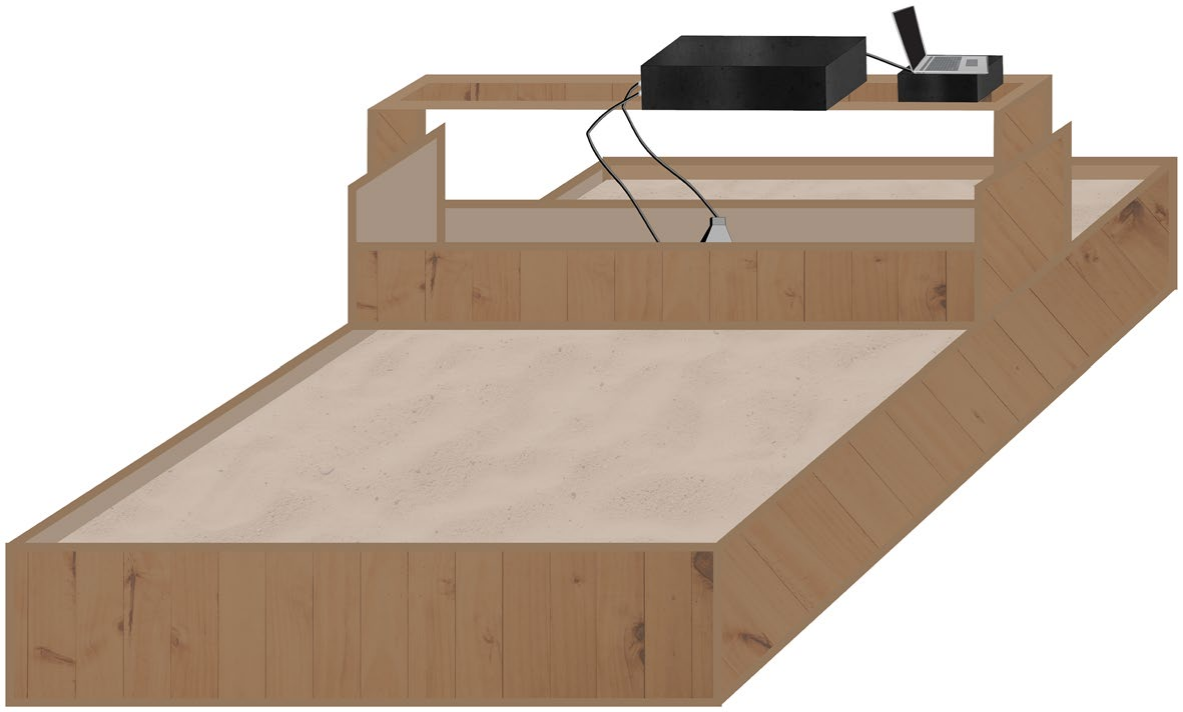
Illustration of the GPR configuration utilized for generating experimental training and testing data used by the proposed surrogate-assisted buried object characterization framework.

For the sake of supplementary demonstration of the proposed surrogate modeling approach, an additional study has been conducted using experimental data (B-scans) collected through the measurements in a “sand pool” environment. A sparse data set is utilized that contains 33 scenarios in total, 27 scenarios for training and the remaining scenarios utilized for testing. It should be emphasized gathering the experimental data is an expensive endeavor due to the considerable manual labor involved (digging and burying targets with high accuracy), as well as the adjustments/maintenance of the measurement system. These are the main reasons for using data-driven surrogate modeling approaches in the field of GPR characterization/detection systems. In this section, our aim is to demonstrate that the proposed technique is also applicable in the case of using physical measurements as a source of data. The experimental samples are obtained in the laboratory at Yıldız Technical University. During the process, raw B-scan data corresponding to various scenarios are generated by the impulse ground penetrating near-zone radar system, which is utilized in various subsurface imaging operations^50–53^. Figure 14 shows the experimental setup. The measurements are taken in a wooden pool filled with dry soil utilized as subsurface. The scanning subsurface domain has the dimensions of 1.90 m (width), 0.22 m (depth) and 1.15 m (length). The cylindrical PEC object is buried in the inhomogeneous dry soil consisting of a mixture of small stones and sand. The experimental setup (GPR, transmitter and receiver antennas) is manually relocated above the subsurface along the scanning path of the approximate length of 1.90 m. Each B-scan is set as 255 (discrete time step) × 95 (A-scan number). The discrete time step is arranged for 6 ns according to the sample rate within 7.5 ns of the compressed data obtained by using gprMax simulation tool. In addition, the measurement data in the form of and *N*_*t*_ × *k* (position number at the scanning aperture) matrix includes 255 × 95 elements. The value of the A-scan number is expressed as 95 and it is defined according to the step size which is 20 mm gives the distance between two consecutive A-scan measurements^9,10,22^.

**Figure 15:**
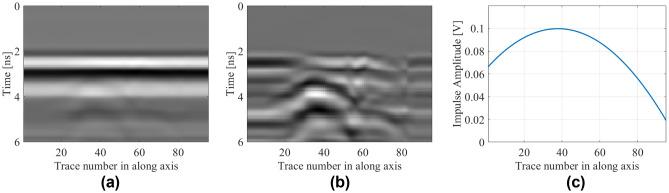
Raw and pre-processed B-scan data with their extracted hyperbolic patterns for selected sample concerning the following test scenario which corresponds to *D* = 750 mm, *P* = 90 mm, and *R* = 15 mm: (**a**) raw B-scan image, (**b**) B-scan image with removed background reflections, (**c**) extracted hyperbolic pattern.

According to the proposed approach, background subtraction is applied to obtained raw B-scan images. Subsequently, hyperbolic signatures are extracted from pre-processed measured data (B-scans) by using the linear regression technique. Figure 15 shows an example scenario with its raw B-scan data, pre-processed B-scan data, and the extracted hyperbolic signature utilized as the input for the purpose of surrogate modeling. By following this approach, 1-D reduced data set is obtained. The extracted hyperbolic pattern, representing the amplitude of the reflected impulse versus the A-scan index along the scanning path is associated with the characteristic parameters of buried object in terms of depth, lateral position and radius.

**Figure 16:**
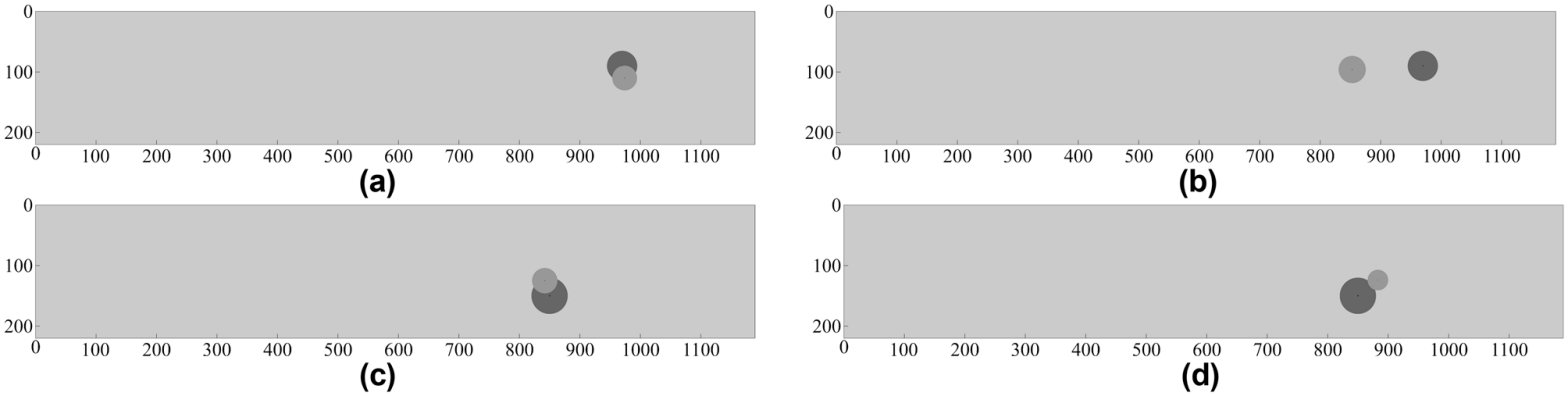
Prediction of geophysical parameters of the object, obtained using the proposed surrogate model and the benchmark model, CNN (using measured data). The target and surrogate-predicted object marked using the dark- and light-grey shade, respectively: Scenario I: *D* = 90 mm, *P* = 970 mm, *R* = 25 mm, (**a**) proposed model, (**b**) CNN; Scenario II: *D* = 150 mm, *P* = 850 mm, *R* = 30 mm, (**c**) proposed model, (**d**) CNN.

Table 11 shows the modeling performance of the proposed surrogate approach and one of the benchmark methods, CNN. The error of the M2LP framework is 27.3 mm (MAE) and 17.9% (RME), which is the average of considered outputs (depth, lateral position and radius). The CNN model exhibits considerably worse accuracy with the average error being almost twice as high. Figure 16 shows the prediction performance for selected scenarios. It should be noted that the proposed method accurately predicts the location of the object (Fig. 16a,c), whereas CNN prediction is poor with this respect (Fig. 16b,d). The prediction of the object size is considerably better for the M2LP model as well.

**Table 11:** Prediction performance of considered characteristic parameters for M2LP and CNN models by using real data set obtained from measurements.

Model	Characteristic	MAE [mm]	RME [%]	Average MAE [mm]
M2LP	Depth	29.3	27.5	27.3
	Lateral position	47.9	5.3	
	Radius	4.5	21.1	
CNN	Depth	18.0	15.2	43.0
	Lateral position	105.9	11.3	
	Radius	5.2	23.0

## Conclusion

This paper introduced a novel regression-assisted surrogate modeling technique for characterization of buried objects. The proposed M2LP framework consists of deep-learning-based feedforward network configuration in the form of a series of blocks of fully connected layers, batch normalization, and activation layers, the latter involving the ReLU activation functions. The B-scan data utilized for object characterization is obtained by using FDTD-based electromagnetic simulation toolbox of a Ground Penetrating Radar (GPR) for estimating of geophysical parameters of a cylindrical shape objects of various radii, buried at different locations. One of the novelties of the work is utilization of the hyperbolic signatures, extracted from the pre-processed B-scans by using linear regression technique, as the inputs of the modeling process. The latter simplified and reduces dimensionality of the input parameter space. This does not only enhance computational efficiency of model construction but also permits a rendition of accurate data-driven surrogates using small number of training data samples. The presented approach is compared to a number of benchmark models including MLP, SVRM, and CNN. The results indicate competitive prediction performance of our method with the average MAE as low as 10 mm versus 18.3 mm for the best benchmark model, CNN. For supplementary verification, the proposed methodology has been applied to buried object characterization using noisy data sets of SNR levels corresponding to 30 dB and 20 dB. The modeling error in these cases corresponds the average MAE of 18.8 mm and 24.5 mm respectively, which is excellent given the scarcity of the datasets. Finally, modeling based on experimental data has been discussed as well, corroborating the relevance of the presented approach. For the considered scenarios, the prediction accuracy of the buried object parameters has been shown to be almost twice as good as the best benchmark method (again, CNN). The numerical and experimental data gathered in the paper is indicative of the competitive accuracy of the introduced methodology, as well as the relevance of the considered algorithmic components, especially dimensionality and problem complexity reduction through delegating the modeling process to hyperbolic signature level. The approach presented in this work can be considered a viable alternative to existing surrogate-assisted techniques for buried object characterization both with respect to accuracy and computational efficiency. On the other hand, the proposed methodology some presents limitations, in particular, characterization objects in different soil media, objects made of different material types, and buried multiple objects. It should be emphasized that the proposed model is expandable. One of possible options, to be considered as a part of the future work, is to enable identification of the objects buried in different soil types, different material types, by adding the mentioned features as extra inputs of the underlying surrogate model.

## Data Availability

The datasets generated during and/or analyzed during the current study are available from the corresponding author on reasonable request.

## References

[1] Daniels, D. J. System design. In *Ground Penetrating Radar*, 2nd ed. 13–36 (The Institution of Electrical Engineers, 2004).

[2] Jol, H. M. Electromagnetic principles of ground penetrating radar. In *Ground Penetrating Radar: Theory and Applications*, 1st ed. 5–17 (Elsevier Science, 2009).

[3] Liu T, Su Y, Huang C (2018). Inversion of ground penetrating radar data based on neural networks. Remote Sens..

[4] Özdemir C, Demirci Ş, Yiğit E, Yılmaz B (2014). A review on migration methods in b-scan ground penetrating radar imaging. Math. Probl. Eng..

[5] Joret A (2018). Design and simulation of horn antenna using CST software for GPR system. J. Phys: Conf. Ser..

[6] Qi, J. et al. Simulation of airborne ground penetrating radar model for detecting underground targets based on CST-MWS. In *2019 Photonics & Electromagnetics Research Symposium - Fall (PIERS - Fall), Xiamen, China*, 1877–1882 10.1109/PIERS-Fall48861.2019.9021621 (2019).

[7] Sharma P, Kumar B, Singh D, Gaba SP (2017). Critical analysis of background subtraction techniques on real GPR data. Def. Sci. J..

[8] Liu H (2020). Detection and localization of rebar in concrete by deep learning using ground penetrating radar. Autom. Constr..

[9] Pasolli E, Melgani F, Donelli M (2009). Automatic analysis of GPR images: A pattern-recognition approach. IEEE Trans. Geosci. Remote Sens..

[10] Ozdemir C, Demirci Ş, Yigit E, Kavak A (2007). A hyperbolic summation method to focus B-scan ground penetrating radar images: An experimental study with a stepped frequency system. Microw. Opt. Technol. Lett..

[11] Dou Q, Wei L, Magee DR, Cohn AG (2017). Real-time hyperbola recognition and fitting in GPR data. IEEE Trans. Geosci. Remote Sens..

[12] Zhang Y, Huston D, Xia T (2016). Underground object characterization based on neural networks for ground penetrating radar data. SPIE Nondestruct. Charact. Monit. Adv. Mater. Aerospace. Civil Infrastructure..

[13] Jin Y, Duan Y (2020). Wavelet scattering network-based machine learning for ground penetrating radar imaging: application in pipeline identification. Remote Sens..

[14] Sakaguchi RT, Morton KD, Collins LM, Torrione PA (2015). Recognizing subsurface target responses in ground penetrating radar data using convolutional neural networks. Proc. SPIE Int. Soc. Opt. Eng..

[15] Moalla M, Frigui H, Karem A, Bouzid A (2020). Application of convolutional and recurrent neural networks for buried threat detection using ground penetrating radar data. IEEE Trans. Geosci. Remote Sens..

[16] Lei W (2020). Underground cylindrical objects detection and diameter identification in GPR B-scans via the CNN-LSTM framework. Electronics.

[17] Kumlu D, Erer I (2018). The multiscale directional neighborhood filter and its application to clutter removal in GPR data. SIViP.

[18] Wang H, Ouyang S, Liu Q, Liao K, Zhou L (2022). Buried target detection method for ground penetrating radar based on deep learning. J. Appl. Remote Sens..

[19] Lei W (2019). Automatic hyperbola detection and fitting in GPR B-scan image. Autom. Constr..

[20] Ahmadi R, Fathianpour N (2017). Estimating geometrical parameters of cylindrical targets detected by ground-penetrating radar using template matching algorithm. Arab. J. Geosci..

[21] Zhang X, Han L, Robinson M, Gallagher A (2021). A gans-based deep learning framework for automatic subsurface object recognition from ground penetrating radar data. IEEE Access..

[22] Pasolli E, Melgani F, Donelli M (2010). Gaussian process approach to buried object size estimation in GPR images. IEEE Geosci. Remote Sens. Lett..

[23] Gharamohammadi A, Shokouhmand A (2020). A robust whitening algorithm to identify buried objects with similar attributes in correlation-based detection. J. Appl. Geophys..

[24] Ozkaya U (2020). GPR B scan image analysis with deep learning methods. Measurement.

[25] Temlioglu E, Erer I (2022). A novel convolutional autoencoder-based clutter removal method for buried threat detection in Ground-Penetrating Radar. IEEE Trans. Geosci. Remote Sens..

[26] Yoldemir B, Mehmet Sezgin M (2019). Peak scatter-based buried object identification using GPR-EMI dual sensor system. Nondestructive Test. Eval..

[27] Giannopoulos A (2005). Modelling ground penetrating radar by GprMax. Constr. Build. Mater..

[28] Warren C, Giannopoulos A, Giannakis I (2016). gprMax: Open source software to simulate electromagnetic wave propagation for ground penetrating radar. Comput. Phys. Commun..

[29] Liu B (2021). GPRInvNet: Deep learning-based ground- penetrating radar data inversion for tunnel linings. IEEE Trans. Geosci. Remote Sens..

[30] Wang J, Liu H, Jiang P, Wang Z, Sui Q, Zhang F (2022). GPRI2Net: A deep-neural-network-based ground penetrating radar data inversion and object identification framework for consecutive and long survey lines. IEEE Trans. Geosci. Remote Sens..

[31] Ji Y (2021). Deep neural network-based permittivity inversions for ground penetrating radar data. IEEE Sens. J..

[32] Giannakis I, Giannopoulos A, Warren C (2021). A machine learning scheme for estimating the diameter of reinforcing bars using ground penetrating radar. IEEE Geosci. Remote Sens. Lett..

[33] Giannakis I, Giannopoulos A, Warren C (2019). A machine learning-based fast-forward solver for ground penetrating radar with application to full-waveform inversion. IEEE Trans. Geosci. Remote Sens..

[34] Chapra, S. C. General linear least-squares and nonlinear regression. In *Applied Numerical Methods with Matlab for Engineers and Scientists*, 4th ed. 385–389. (Mc Graw Hill Education, 2018).

[35] Jaluria, Y. Numerical curve fitting and interpolation. In *Computer Methods for Engineering with Matlab Applications*, 2nd ed. 252–257 (CRC Press Taylor & Francis Group, 2011).

[36] Yurt, R., & Torpi, H. Ground penetrating radar data analysis with nonlinear regression on artificial neural network. In *2020 International Congress on Human-Computer Interaction, Optimization and Robotic Applications (HORA)*, 1–5, (2020).

[37] Calik N, Belen MA, Mahouti P (2020). Deep learning base modified MLP model for precise scattering parameter prediction of capacitive feed antenna. Int J. Numer. Model..

[38] Kumar A, Singh UK, Pradhan B (2022). Ground penetrating radar in coastal hazard mitigation studies using deep convolutional neural networks. Remote Sens..

[39] Barkataki N, Tiru B, Sarma U (2022). A CNN model for predicting size of buried objects from GPR B-scans. J. Appl. Geophys..

[40] Giannakis I, Zhou F, Warren C, Giannopoulos A (2022). On the limitations of hyperbola fitting for estimating the radius of cylindrical targets in nondestructive testing and utility detection. IEEE Geosci. Remote Sens. Lett..

[41] Kingma, D. P., Ba, J. Adam: A method for stochastic optimization. arXiv:1412.6980v9, (2017).

[42] Ioffe, S. & Szegedy, C. Batch normalization: Accelerating deep network training by reducing internal covariate shift. CoRR, abs/1502.03167(1), 1–11, arXiv:1502.03167, (2015).

[43] Helton JC, Davis FJ (2003). Latin hypercube sampling and the propagation of uncertainty in analyses of complex systems. Rel. Eng. Syst. Saf..

[44] Hosseinzadeh S, Shaghaghi M (2020). GPR data regression and clustering by the fuzzy support vector machine and regression. Prog. Electromagn. Res. M..

[45] Mahouti P (2019). Design optimization of a pattern reconfigurable microstrip antenna using differential evolution and 3D EM simulation-based neural network model. Int. J. RF. Microw. Comput. Aided Eng..

[46] Smitha N, Singh V (2020). Target detection using supervised machine learning algorithms for GPR Data. Sens. Imaging..

[47] Zhu J, Xue W, Rong X, Yu Y (2017). A clutter suppression method based on improved principal component selection rule for ground penetrating radar. Prog.Electromagn. Res. M..

[48] Shehab MA (2019). Subspace clutter removal techniques in GPR images. Prog. Electromagn. Res. M..

[49] Yurt R, Torpi H, Mahouti P, Kızılay A, Koziel S (2023). Buried object characterization using ground penetrating radar assisted by data-driven surrogate-models. IEEE Access..

[50] Turk, A.S. UWB performance analysis of PDTEM horn antenna designed for multi-sensor adaptive hand-held GPR. In *11th International Symposium on Antenna Technology and Applied Electromagnetics [ANTEM 2005]*. 1–4, (2005).

[51] Turk AS (2004). Ultra-wideband TEM horn design for ground penetrating impulse radar systems. Microw. Opt. Technol. Lett..

[52] Turk AS, Aksoy S, Keskin AK, Senturk MD, Caliskan A, Ozakin MB (2015). Ultra-wide band antenna designs and numerical system modelling for forward-looking GPR. Near Surf. Geophys..

[53] Turk AS, Keskin AK, Senturk MD (2015). Dielectric loaded TEM horn-fed ridged horn antenna design for ultrawideband ground-penetrating impulse radar. Turk. J. Electr. Eng. Comput. Sci..

